# The dipeptidyl peptidase IV inhibitors vildagliptin and K-579 inhibit a phospholipase C: a case of promiscuous scaffolds in proteins

**DOI:** 10.12688/f1000research.2-286.v3

**Published:** 2015-01-29

**Authors:** Sandeep Chakraborty, Adela Rendón-Ramírez, Bjarni Ásgeirsson, Mouparna Dutta, Anindya S. Ghosh, Masataka Oda, Ravindra Venkatramani, Basuthkar J. Rao, Abhaya M. Dandekar, Félix M. Goñi

**Affiliations:** 1 Department of Biological Sciences, Tata Institute of Fundamental Research, Mumbai, 400 005, India; 2 Plant Sciences Department, University of California, Davis, CA, 95616, USA; 3 Unidad de Bio, Universidad del Pais Vasco, Bilbao, Spain; 4 Science Institute, Department of Biochemistry, University of Iceland, IS-107 Reykjavik, Iceland; 5 Department of Biotechnology, Indian Institute of Technology Kharagpur, Kharagpur, 721302, India; 6 Division of Microbiology and Infectious Diseases, Niigata University Graduate School of Medical and Dental Sciences, Niigata, 951-8514, Japan; 7 Department of Chemical Sciences, Tata Institute of Fundamental Research, Mumbai, 400 005, India

**Keywords:** diabetes, Bacillus cereus, PI-PLC, serine protease, lipase

## Abstract

The long term side effects of any newly introduced drug is a subject of intense research, and often raging controversies. One such example is the dipeptidyl peptidase-IV (DPP4) inhibitor used for treating type 2 diabetes, which is inconclusively implicated in increased susceptibility to acute pancreatitis. Previously, based on a computational analysis of the spatial and electrostatic properties of active site residues, we have demonstrated that phosphoinositide-specific phospholipase C (PI-PLC) from *Bacillus cereus* is a prolyl peptidase using *in vivo* experiments. In the current work, we first report the inhibition of the native activity of PI-PLC by two DPP4 inhibitors - vildagliptin (LAF-237) and K-579. While vildagliptin inhibited PI-PLC at micromolar concentrations, K-579 was a potent inhibitor even at nanomolar concentrations. Subsequently, we queried a comprehensive, non-redundant set of 5000 human proteins (50% similarity cutoff) with known structures using serine protease (SPASE) motifs derived from trypsin and DPP4. A pancreatic lipase and a gastric lipase are among the proteins that are identified as proteins having promiscuous SPASE scaffolds that could interact with DPP4 inhibitors. The presence of such scaffolds in human lipases is expected since they share the same catalytic mechanism with PI-PLC. However our methodology also detects other proteins, often with a completely different enzymatic mechanism, that have significantly congruent domains with the SPASE motifs. The reported elevated levels of serum lipase, although contested, could be rationalized by inhibition of lipases reported here. In an effort to further our understanding of the spatial and electrostatic basis of DPP4 inhibitors, we have also done a comprehensive analysis of all 76 known DPP4 structures liganded to inhibitors till date. Also, the methodology presented here can be easily adopted for other drugs, and provide the first line of filtering in the identification of pathways that might be inadvertently affected due to promiscuous scaffolds in proteins.

## Introduction

Oral glucose elicits a greater insulin response than intravenous glucose infusion, a phenomenon known as the incretin effect ^1^. This effect is mostly attributed to the intestinally derived hormones glucagon-like peptide-1 (GLP-1) and gastric inhibitory polypeptide (GIP) ^2^. These hormones have a very short half-life as they are rapidly inactivated by the ubiquitous enzyme dipeptidyl peptidase-IV (DPP4) ^3^. The finding that the incretin effect is impaired in subjects with type 2 diabetes ^4^ led to two major types of GLP-1 based therapies ^5^ - intravenously or sub-cutaneously administered GLP-1 mimetics that are resistant to DPP4 (exenatide, liraglutide, etc.) ^6^, and the orally administered gliptins that prolong the physiological actions of incretin hormones by inhibiting DPP4 (sitagliptin, vildagliptin, etc.) ^7– 9^. Due to the multifarious roles played by the DPP4 enzyme ^10– 12^, the possible side effects of these drugs (acute pancreatitis, pancreatic cancer, etc. ^13– 15^) are strongly contested by researchers who argue that current statistics are insufficient ^16, 17^ to conclusively attribute these side effects to the otherwise beneficial GLP-1 drugs ^18^. Compound promiscuity is another phenomenon that might play a crucial role in determining the side effects of these therapies, although this aspect has rarely been pursued intensively ^19^.

Previous work by our group has established the spatial and electrostatic congruence in cognate residue pairs of the active site in proteins with the same functionality (CLASP) ^20, 21^. CLASP analysis indicated that the phosphoinositide-specific phospholipase C (PI-PLC) from *Bacillus cereus* has spatial and electrostatic congruence with a serine protease motif ^22^. This was validated by protease assays, mass spectrometry and by inhibition of the native phospholipase activity of PI-PLC by the well-known serine protease inhibitor AEBSF (IC _50_ = 0.018 mM). The specificity of the protease activity was for a proline in the amino terminal, suggesting that PI-PLC is a prolyl peptidase, similar to the DPP4 enzyme. This finding led us to believe that the gliptins would have similar inhibitory effect on PI-PLC. In the current work, we have confirmed the inhibition of the native phospholipase activity of PI-PLC using two gliptins - vildagliptin ^23^ (at *µ*-molar concentrations) and K579 ^24^ (at nano-molar concentrations).

Subsequently, we used a motif derived from a DPP4 protein ^25^, in addition to the trypsin motif used previously ^22^, to query a comprehensive and non-redundant (50% sequence identity) list of ~5000 human proteins with known structures using CLASP, intending to identify other proteins that might be inhibited by the gliptins. From the set of proteins with significant congruent matches with these two motifs, we identified a pancreatic lipase ^26^ and a gastric lipase ^27^, keeping the context of lipases, acute pancreatitis and GLP-1 based therapies in mind. Our findings rationalize the elevated levels of serum lipase found in patients undergoing DPP4 inhibitor based therapies ^28, 29^, although these reports are in disagreement with other findings ^30, 31^. While it is logical and expected to find scaffolds that are congruent to trypsin and DPP4 active sites in lipases based on the current results and our previous findings ^22^, we also show the presence of the serine catalytic triad in close proximity to the active site residues of proteins which have a completely different enzymatic mechanism (for example, in glutaminyl cyclase which is a transferase ^32^). This corroborates the current belief that convergent evolution occurs more frequently than previously believed ^33^. Thus, we propose a rational method to identify proteins that might have unintended and undesirable interactions with newly introduced compounds, and substantiate our claims by demonstrating the inhibition of the native phospholipase activity of PI-PLC from *B. cereus* using gliptins that are used in type 2 diabetes therapy.

## Results

### The active site motifs

The active sites of serine proteases differ in their specificities owing to residues other than the conserved catalytic triad. Thus, in addition to the trypsin motif used previously (Asp102, Ser195 and His57 - PDBid 1A0J) ^22^ (Motif1), we choose another motif from a DPP4 enzyme (Asp708, Ser630 and His740 - PDBid:1N1M) (Motif2) ( Table 1). Apart from the catalytic triad, we chose another non-polar residue in order to increase the specificity of the matches (Ala56 in Motif1 and Val711 in Motif2). This fourth residue is chosen as the closest residue to any one of the catalytic triad residues. Using the ability of CLASP to include stereochemically equivalent residues, this last residue could be matched by another non-polar residue - one of Gly, Ala, Val, Leu, Ile or Met. Further, it has been seen that the second (ac) and fifth (bd) ( Table 1) pairwise electrostatic potential differences (EPD) are not discriminatory - thus, this pair is not used to score the EPD difference (although it is included in the distance deviation score).

**Table 1.** Potential and spatial congruence of the active site residues in proteins queried using two motifs - Motif1 from Trypsin and Motif2 from DPP4. Rmsd1 and Rmsd2 are the root mean square deviation of the scaffold with respect to Motif1 and Motif2. DPP4 - dipeptidyl peptidase-IV, PI-PLC - phosphoinositide-specific phospholipase C, PLASE - human pancreatic lipase-Related Protein 2, GPASE - human gastric lipase, QC - glutaminyl cyclase. D = Pairwise distance in Å. PD = Pairwise potential difference. APBS writes out the electrostatic potential in dimensionless units of kT/e where k is Boltzmann’s constant, T is the temperature in K and e is the charge of an electron.

PDB	Active site atoms (a,b,c,d)		ab	ac	ad	bc	bd	cd	Rmsd1	Rmsd2
TRYPSIN (1A0J)	D102,S195 H57,A56	D PD	7.8 -144.1	5.6 -39.2	2.9 -248.3	3.3 104.8	9.0 -104.3	6.9 -209.1	0	0.5
DPP4 (1N1M)	D708,S630 H740,V711	D PD	7.6 -154.4	5.4 124.4	2.6 -148.8	2.6 278.8	6.8 5.6	5.4 -273.2	0.5	0
PI-PLC (1PTD)	D67,S234 H32,I68	D PD	8.2 -93.7	6.2 39.7	4.1 -245.2	3.8 133.4	11.5 -151.5	9.2 -284.8	0.6	1.1
PLASE (2OXE)	D195,S171 H282,G235	D PD	7.7 -150.2	6.4 26.7	4.4 -132.1	3.0 176.9	6.7 18.2	5.8 -158.8	0.5	0.4
GPASE (1HLG) Motif1	D324,S153, H353,L326	D PD	7.5 -202.6	5.0 -15.0	2.9 -272.3	2.7 187.6	8.4 -69.7	6.2 -257.3	0.2	0.3
GPASE (1HLG) Motif2	D324,S153 H353,A327	D PD	7.5 -202.6	5.0 -15.0	2.6 -207.1	2.7 187.6	7.1 -4.5	5.3 -192.1	0.4	0.1
QC (3PB4)	D170,S187, H168,G224	D PD	7.5 -92.8	4.8 -16.5	3.4 -214.0	3.3 76.3	10.7 -121.2	8.0 -197.5	0.4	0.8

**Table 2.** Best matches in the set of ~5000 human proteins. (a) Motif1 (Asp102, Ser195, His57, Ala56) from Trypsin (b) Motif2 (Asp708, Ser630, His740, Val711) from DPP4.

Motif	PDB	Description	CLASP Score
1	2ANY	Plasma kallikrein, light chain	0.028
1	2OQ5	Transmembrane protease, serine 11E	0.037
1	3U0V	Lysophospholipase-like protein 1	0.041
1	2ODP	Complement C2	0.060
1	1IMJ	CCG1-interacting factor B	0.065
1	3F6U	Vitamin K-dependent protein C heavy chain	0.065
1	1ELV	Complement C1S component	0.068
1	1MD8	C1R complement serine protease	0.068
1	1ORF	Granzyme A	0.070
1	1FJ2	Acyl protein thioesterase 1	0.071
2	1HLG	Gastric lipase	0.042
2	1SPJ	Kallikrein 1	0.114
2	2F83	Coagulation factor XI	0.120
2	1ZJK	Mannan-binding lectin serine protease 2	0.131
2	3QLP	Thrombin light chain	0.145
2	2QXI	Kallikrein-7	0.146
2	2XU7	Histone-binding protein RBBP4	0.174
2	2W2N	Proprotein convertase subtilisin/kexin type 9	0.180
2	2HEH	KIF2C protein	0.195
2	2ANY	Plasma kallikrein, light chain	0.197

**Figure 1.**
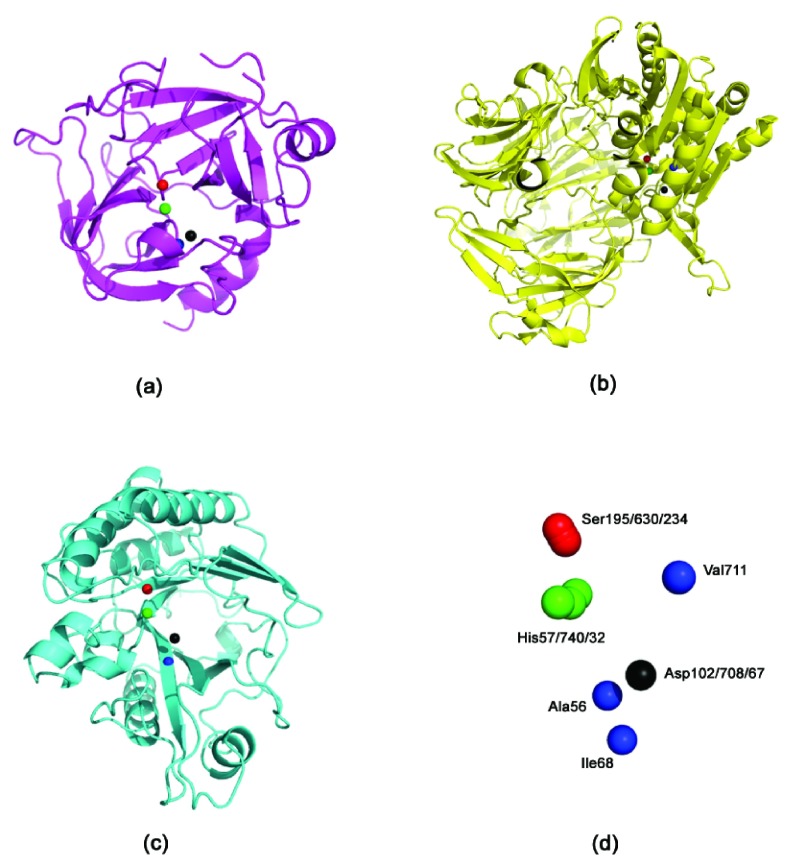
The active site residues in Trypsin, DPP4 and PI-PLC. ( **a**) Trypsin (PDBid:1A0J) ( **b**) DPP4 (PDBid:1N1M); ( **c**) PI-PLC (PDBid:1PTD) ( **d**) Superimposing the active site residues using DE- CAAF ^35^. The superimposition can be viewed in Superimposeproteins.p1m in Dataset 1.

**Figure 2.**
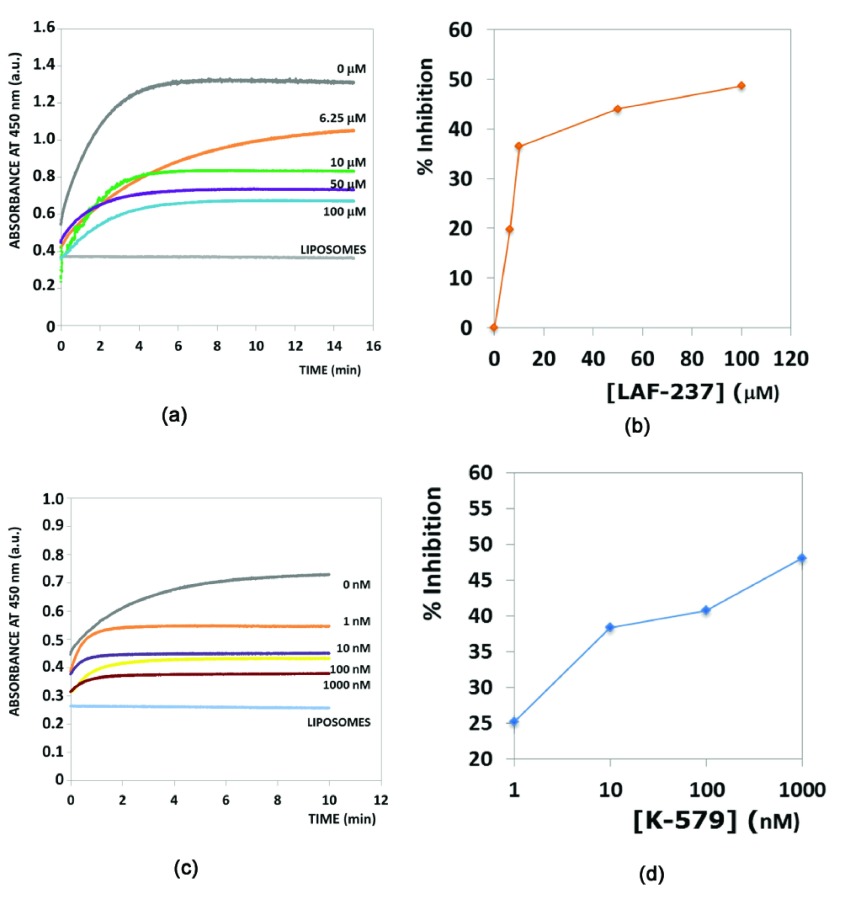
PI-PLC inhibition using DPP4 inhibitors. ( **a**, **c**) Time courses of enzyme activity in the presence of varying amounts of inhibitors, respectively LAF-237 and K579. The trace marked LIPOSOMES corresponds to a control in the absence of PI-PLC. ( **b**, **d**) Dose-response effect of inhibitors on PI-PLC activity. Activity was computed as the extent of vesicle aggregation after 10 min enzyme activity.

**Supplementary Figure 1.**
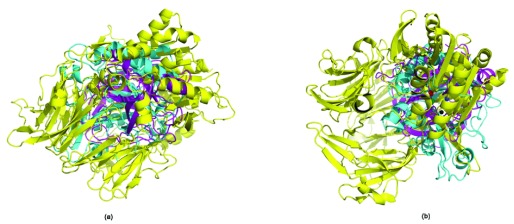
Superimposition of trypsin (PDBid:1A0J - magenta), dipeptidyl peptidase-IV (PDBid:1N1M - yellow) and phosphoinositide-specific phospholipase C (PDBid:1PTD - cyan). It is seen that there is no structural similarity in the two proteins. ( **a**) Using MUSTANG ^34^. ( **b**) Using DECAAF ^35^.

***Inhibition of phosphoinositide-specific phospholipase C (PI-PLC) using dipeptidyl peptidase-IV (DPP4) inhibitors.*** DPP4 (EC 3.4.14.5), a serine protease that is expressed in many tissues (kidney, liver, lung, intestinal membranes, lymphocytes and endothelial cells), cleaves peptides with Pro or Ala residues in the second amino terminal position. Previously, we have experimentally demonstrated the existence of the serine protease domain in PI-PLC from *Bacillus cereus* - both by virtue of its proteolytic activity, and the inhibition of its native activity on phospholipids in the presence of serine protease inhibitors ^22^. Furthermore, the specificity of the proteolytic activity indicated that it was a prolyl peptidase - thus, leading us to believe that DPP4 inhibitors should have a similar inhibitory effect on the PI-PLC enzyme. Table 1 shows the presence of a congruent motif in the PI-PLC protein with both Motif1 and Motif2. His32 and Asp67 are known to be a part of the active site scaffold in PI-PLC ^22^. These proteins have completely different folds, and thus a superimposition (using both MUSTANG ^34^ and DECAAF ^35^) does not show any detectable similarity in their structures ( Supplementary Figure 1). Figure 1 shows the active sites of these proteins, and the superimposition of these proteins based on their catalytic residues ^35^. It can be seen that the closest non-polar residue to the catalytic triad in trypsin and PI-PLC (Ala56 in PDBid:1A0J, Ile68 in PDBid:1PTD) is differently placed from Val711 in DPP4 (PDBid:1N1M). This is also indicated by the greater RMSD (root mean square deviation) of the scaffold in PI-PLC to Motif2 as compared to Motif1. The differences in the position of peripheral residues is the source of the diverse specificities exhibited by these proteases. Figure 2 shows the inhibition of PI-PLC using two gliptins - vildagliptin (LAF-237) ^23^ and K579 ^24^. PI-PLC catalyzes hydrolysis of phospholipids to yield diacylglycerol and a phosphoryl alcohol. In the absence of inhibitors enzyme addition to the vesicle suspension causes an increase in turbidity due to vesicle aggregation ( Figure 2 a,c). Aggregation in turn occurs as a result of formation of the enzyme endproduct diacylglycerol ^36, 37^. A steady-state is reached under our conditions after 6–8 min. Addition of either LAF-237 (vildagliptin) or K579 leads to an obvious inhibition of the enzyme activity. Dose-response curves for the inhibitors are shown in Figure 2 (b,d). K579 is two orders of magnitude more potent than LAF-237 as a PI-PLC inhibitor, with half-maximal inhibitory concentrations IC _50_ respectively of 1 *µ*M and 100 *µ*M.

Phosphoinositide-specific phospholipase C inhibition data using the dipeptidyl peptidase-IV inhibitors K-579 and LAF-237 Data set for Fig 2 in the main article, describing the inhibition of Phosphoinositide-specific phospholipase C inhibition using the dipeptidyl peptidase-IV inhibitors K-579 and LAF-237.

***Querying a non-redundant set of human proteins using Motif1 and Motif2.*** Currently, the PDB database has about 25,000 human proteins. Using a identity cutoff of 50%, we chose a set of ~5000 proteins ( Supplementary Table 1) as the target proteins. Table 2 shows ten proteins which have signicant matches with Motif1 and Motif2. Given the context of lipases, acute pancreatitis and GLP-1 based therapies, we picked two proteins - the human pancreatic lipase-related protein 2 (PDBid:2OXE) ^26^ and a human gastric lipase (PDBid:1HLG) ^27^ - to demonstrate the distinct possibility that these proteins might be inhibited by DPP4 inhibitors. Table 1 shows the congruence of the DPP4 motif to these proteins using Motif1 and Motif2. It is interesting to note that the gastric lipase (PDBid:1HLG) has a good match with both motifs - Leu326 in PDBid:1HLG is congruent to Ala56 in PDBid:1A0J, and Ala237 (PDBid:1HLG) is congruent to Val711 (PDBid:1N1M).

**Figure 3.**
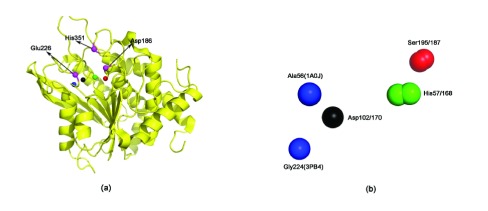
A scaffold congruent to the active site of Trypsin (PDBid:1A0J) in a glutaminyl cyclase (PDBid:3PB4). ( **a**) The active site residues are marked in magenta. They are seen to be proximal to the identified scaffold. ( **b**) Superimposition of Motif1 and the scaffold in glutaminyl cyclase. The exact pairwise interatomic distance and electrostatic potential differences are specified in Table 1.

Since both these proteins are lipases (hydrolases), this congruence to Motif1 and Motif2 is expected based on our previous results with PI-PLC ^22^. However, our methodology also detects other proteins, often with a completely different enzymatic mechanism from hydrolases. A glutaminyl cyclase (PDBid:3PB4 ^32^), a transferase, has a significantly congruent domain with Motif1 (lesser congruence with Motif2, as indicated by the RMSD) ( Table 1). Figure 3 shows the proximity of the promiscuous scaffold to the active site of the cyclase, and also the congruence of the scaffold to Motif1.

**Figure 4.**
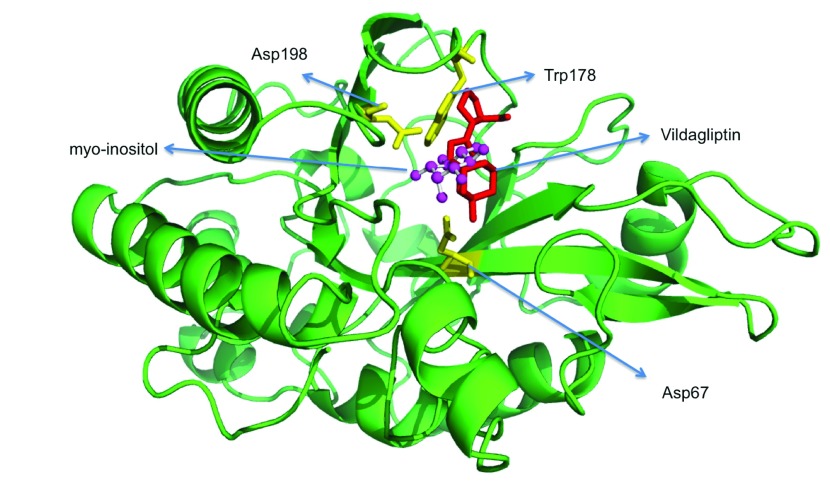
Docking vildagliptin to the PI-PLC structure in complex with myo-inositol (PDBid:1PTGA). Docking done using DOCLASP ^40^. The Pymol script for visualizing the docking (SupplementaryPymol.p1m) is provided as Supplementary information.

***Docking vildagliptin to the PIPLC structure.*** Since there are no DPP4 structures solved which ligand K-579, a DPP4 protein structure in complex with vildagliptin (PDBid:3W2TA) ^38^ was used to dock vildagliptin to the PIPLC structure complexed with myo-inositol (PDBid:1PTG ^39^) using DOCLASP ^40^ ( Figure 4). The Pymol script for visualizing the docking (SupplementaryPymol.p1m) is provided as Supplementary information.

***Statistics of atoms making contact with inhibitors.*** There are 76 unique DPP4 inhibitors, defined by three letter codes, for which the ligand-DPP4 structure is solved ( Supplementary Table 2). For uniformity, we chose the first four closest atoms from the protein that make contacts to the ligand, excluding hydrophobic interactions. Table 3 shows the number of times each residue in DPP4 makes contact to the ligand. Three residues are ubiquitous in making contacts in all these ligands: Glu205, Glu206 and Tyr662 made contacts in 71, 68 and 63 ligands, respectively. Interestingly, Glu205 and Glu206 have been implicated as critical residues for the enzymatic activity of DPP4 through point mutations ^41^. Note, that since only the first four residues were considered, these counts are conservative (and might be more). A recent study has found that inhibitors that bind to residues beyond the extensive subsite (defined as Val207, Ser209, Phe357 and Arg358) increases DPP4 inhibition, as compared to those inhibitors that form a covalent bond with Ser630 ^38^. Table 3 shows that very few inhibitors make such contacts. We created a library of motifs from these structures that can be used to query any protein using CLASP to determine the possibility that DPP4 inhibitors might bind to it ( Supplementary Table 3), after removing equivalent ones to eliminate redundancy. This table shows the final list of 39 motifs (pruned from the initial 76): this is a comprehensive set of motifs that encapsulates the current knowledge about protein ligand interactions for the DPP4 enzyme. A facet of ligand binding that needs to be accounted for while choosing a motif is the spatial and electrostatic changes that can be induced by ligand binding. Thus, we obtain the residues involved in binding from the holo enzyme, but extract the motif values (pairwise distance and EPD) from the apo enzyme.

**Table 3.** Number of times residues from the DPP4 enzyme ligand an inhibitor. Three residues - Glu205, Glu206 and Tyr662 - make contacts in 71, 68 and 63 ligands, respectively. Note, that since we only choose the first four residues based on proximity of the atoms closest to the ligand, these counts are conservative (and might be actually more).

Residue	Number of ligands
ARG125	11
GLU205	71
GLU206	68
VAL207	1
SER209	3
ARG358	6
TYR547	18
GLN553	1
TYR585	1
TRP629	1
SER630	10
TYR631	12
TYR662	63
ASN710	15

## Discussion

The controversy regarding the side effects of the dpp4 inhibitors, particularly with respect to acute pancreatitis and pancreatic cancer, continues unabated. While some researchers feel that it is not acceptable to assume that ‘absence of evidence is evidence of absence’ ^42, 43^, others believe that current data are not conclusive and the ‘benefits by far outweigh the potential risks’ ^16^. Adding to the uncertainties are conflicting reports presented by different groups ^28– 31^. Notwithstanding the antagonistic views on the subject, it is unanimously accepted that current data are insufficient to establish a causal pathogenic effect of these drugs on such side effects ^44^.

Various database studies have been undertaken in order to ascertain the effects of the GLP-1 therapies. Some studies ‘did not find an association between the use of exenatide or sitagliptin and acute pancreatitis’ with the caveat that the ‘limitations of this observational claims-based analysis cannot exclude the possibility of an increased risk’ ^45^. On the other hand, other studies have shown that the use of ‘sitagliptin or exenatide increased the odds ratio for reported pancreatitis 6-fold as compared with other therapies’ ^14^. Further, they reported that ‘pancreatic cancer was more commonly reported among patients who took sitagliptin or exenatide as compared with other therapies’ ^14^. Although these studies concern the usage of both GLP-1 mimetics and the orally administered gliptins, and our study exclusively focusses on gliptins, and is not concerned with the GLP-1 mimetics data. The close relationship between chronic pancreatitis and pancreatic cancer is also a subject of intense research ^46^. Another administrative database study of US adults with type 2 diabetes reported increased odds of hospitalization for acute pancreatitis for patients undergoing GLP-1 based therapies sitagliptin ^13^. Once again, such correlation of GLP-1 based therapies to acute pancreatitis is contested by other studies ^47^.

Our findings rationalize the elevated levels of serum lipase found in patients undergoing DPP4 inhibitor based therapies ^28, 29^, keeping in mind that other studies contradict these reports ^30, 31^. While several studies have reported that the GLP-1 mimetics do not induce pancreatitis in rats, mouse and/or monkey ^48– 50^, these studies did not include DPP4 inhibitors, which are the compounds that might be responsible for interactions with pancreatic proteins according to our study. It is to be noted however that these mimetics may have other physiological effects and ‘the long-term consequences of sustained GLP-1 receptor activation in the human thyroid remain unknown and merit further investigation’ ^51^. Once again, the previous study ^51^ has been challenged by another group who note that ‘findings previously reported in rodents may not apply to humans’ ^52^.

The orally administered gliptins differ in many aspects such as potency, excretion mechanism, target selectivity, half-life, metabolism and possible drug-drug interactions ^9, 53, 54^. This difference is also highlighted in the different concentrations of vildagliptin and K579 that inhibit PI-PLC. A recent study has also noted the differential off-target inhibition of enzymes by vildagliptin and sitagliptin using a high-throughput, multiplexed assay ^55^. Interestingly, the PI-PLC scaffold has a better match with the trypsin motif than with the DPP4 motif ( Table 1). In order to be able to model these differences in our *in silico* search, it is important to be able to provide flexibility in the scoring mechanism.

To summarize, it has been noted in the case of GLP-1 based therapies that as ‘evidence of harm accumulates, but is vigorously discounted’ the ‘burden of proof now rests with those who wish to convince us of their safety’ ^43^. Surveillance programs, real-life cohort studies and case-control studies can be supplemented by rational investigations of relevant proteins based on anecdotal reports ^56^. The methodology proposed in the current work, which specifically demonstrates the effects of the DPP4 inhibitors, also presents a rational way of determining the inadvertent interactions of newly designed compounds with proteins, and thus prevent the recurrence of drug induced diseases being detected after considerable damage has already been inflicted on humans subjected to these drugs ^57^.

## Materials and methods

### *In silico* analysis

A comprehensive, non-redundant set of ~5000 human proteins (50% identity cutoff) was obtained from the PDB database ^58^. The CLASP package ( http://www.sanchak.com/clasp) used for querying these proteins using motifs from trypsin and DPP4 is written in Perl on Ubuntu ^20^. Hardware requirements are modest - all results here are from a simple workstation (8GB ram), and runtimes for analyzing the ~5000 proteins was about 24 hours. Adaptive Poisson-Boltzmann Solver (APBS) and PDB2PQR packages were used to calculate the potential difference between the reactive atoms of the corresponding proteins ^59, 60^. The APBS parameters and electrostatic potential units were set as described previously in Chakraborty *et al.* ^20^. All protein structures were rendered by PyMol ( http://www.pymol.org/). Protein structures have been superimposed using MUSTANG ^34^ and DECAAF ^35^.

### Protein, substrate and reagents

PI-PLC was purchased from Sigma. Vildagliptin (LAF-237) was obtained from Selleckchem, and K579 was obtained from Santa Cruz.

### PI-PLC assay and inhibition using DPP4 inhibitors

***Vesicle preparation and characterization.*** The appropriate lipids were mixed in organic solution, and the solvent was evaporated to dryness under N _2_. Solvent traces were removed by evacuating the lipids for at least 2 hours. The lipids were then swollen in 10 mM Hepes, 150 mM NaCl, pH 7.5 buffer. Large unilamellar vesicles (LUV) were prepared from the swollen lipids by extrusion and sized by using 0.1 *µ*m poresize Nuclepore filters, as described by Ahyayauch *et al.* ^36^. LUV composition was egg phosphatidylcholine: egg phosphatidylethanolamine: cholesterol at a 2:1:1 mole ratio. The average size of LUV was measured by quasi-elastic light scattering, using a Malvern Zeta-sizer instrument. Lipid concentration, determined by phosphate analysis, was 0.3 mM in all experiments.

***Aggregation Assay.*** Enzyme activity was assayed measuring enzyme-induced vesicle aggregation. All assays were carried out at 39°C with continuous stirring, in 10 mM Hepes, 150 mM NaCl buffer (pH 7.5), in the presence of 0.1% BSA for optimum catalytic activity. Enzyme concentration was 0.16 U/mL, and liposomal concentration was 0.3 mM. Lipid aggregation was monitored in a Cary Varian UV-vesicle spectrometer as an increase in turbidity (absorbance at 450 nm) of the sample, as described by Villar *et al.* ^37^. The data are average values of two closely similar experiments.

***Analyzing known DPP4 inhibitors with solved structures.*** In order to obtain all known structures of DPP4 with inhibitors bound to the active site, we did a search for the keyword dipeptidyl-peptidase on the PDB database, and choose proteins with DPP4 inhibitors as ligands. There are 76 such unique compounds (defined by three letter codes) that are reported to date (May 2014). We docked the DPP4 inhibitor to the PIPLC active site using DOCLASP ^40^.

## Data availability

figshare: Phosphoinositide-specific phospholipase C inhibition data using the dipeptidyl peptidase-IV inhibitors K-579 and LAF-237, http://dx.doi.org/10.6084/m9.figshare.880620

## Figures and Tables

**Supplementary Table 1.** PDB IDs of ∼5000 human proteins analyzed in this study.

1A02 1A12 1A17 1A1W 1A1X 1A27 1A31 1A35 1A36 1A3Q 1A4I 1A66 1A6Q 1A6Y 1A7S 1A9N 1AAP 1AB2 1AD6 1ADX 1ADZ 1AIE 1AJJ 1ALE 1ALF 1ALU 1ALY 1AM9 1AN4 1AOA 1APJ 1APQ 1APY 1AQC 1ATZ 1AU1 1AUD 1AV1 1AVO 1AW0 1AWE 1AWW 1AX8 1AYE 1B1C 1B22 1B34 1B4F 1B4G 1B4Q 1B4R 1B64 1B6A 1B6E 1B6U 1B72 1B9O 1BA5 1BAK 1BBO 1BC7 1BC8 1BCI 1BD8 1BF5 1BH7 1BH9 1BHD 1BHI 1BIK 1BJ8 1BKR 1BL1 1BNK 1BNL 1BO9 1BOE 1BOR 1BPV 1BPX 1BPY 1BPZ 1BQU 1BTK 1BTR 1BTS 1BUO 1BV8 1BW6 1BX4 1BY1 1BY2 1BY4 1BYG 1BYW 1BZ4 1BZG 1C07 1C7U 1C9B 1CB0 1CCZ 1CDW 1CF7 1CI4 1CJY 1CK7 1CKS 1CMZ 1CNT 1CQ0 1CQT 1CR8 1CS8 1CSK 1CSY 1CVJ 1CXW 1CY5 1CZA 1CZT 1CZY 1D2J 1D2L 1D2S 1D3B 1D4A 1D4B 1D4T 1D4U 1D5R 1D7Q 1D8J 1D9N 1DBH 1DD1 1DDJ 1DE8 1DE9 1DEB 1DEU 1DEW 1DG6 1DGF 1DGN 1DHS 1DJL 1DK8 1DLE 1DN3 1DNG 1DNU 1DQB 1DSZ 1DT9 1DTJ 1DUX 1DV0 1DV8 1DXS 1DXX 1DYN 1DZ5 1E07 1E17 1E2S 1E3O 1E4U 1E7K 1E88 1E8O 1E8S 1E8Y 1E9K 1EAJ 1EAZ 1EBM 1EC6 1EDM 1EDN 1EDP 1EER 1EF7 1EFV 1EG3 1EGD 1EGI 1EGT 1EGW 1EH6 1EIG 1EJ9 1EJF 1EK6 1ELK 1ELR 1ELV 1ELW 1EM2 1EMH 1EMJ 1EMN 1EMU 1EQX 1ET1 1ETE 1EVS 1EWF 1EWN 1EX0 1EXT 1EXZ 1EYB 1F05 1F0Y 1F2L 1F2Q 1F3U 1F4R 1F5N 1F5Y 1F62 1F6O 1F6W 1F7E 1F86 1F9P 1FAC 1FAO 1FAQ 1FB1 1FCH 1FCY 1FEW 1FGD 1FGE 1FGU 1FHT 1FIT 1FJ2 1FL0 1FLK 1FM5 1FMK 1FN7 1FNF 1FO1 1FO3 1FOS 1FP0 1FPZ 1FQQ 1FS1 1FSB 1FSU 1FVR 1FW1 1FXL 1FYC 1FYH 1FYJ 1FYV 1FYX 1FZD 1FZV 1G1C 1G1T 1G25 1G2E 1G2S 1G3M 1G55 1G5V 1G84 1G8I 1G8Q 1G91 1G9L 1GA5 1GEN 1GH2 1GJJ 1GJZ 1GK4 1GK7 1GKG 1GKN 1GNA 1GNB 1GO5 1GP0 1GPZ 1GQV 1GR3 1GRI 1GS9 1GTW 1GU4 1GU5 1GVJ 1GW3 1GXC 1GXE 1GXR 1GY5 1H03 1H0Z 1H2I 1H2K 1H30 1H3I 1H3O 1H4A 1H4R 1H5P 1H6G 1H6H 1H6I 1H6K 1H6P 1H7C 1H7S 1H8U 1H95 1H9D 1H9E 1H9F 1H9O 1HA4 1HAE 1HAO 1HAP 1HBX 1HCC 1HCI 1HCN 1HCP 1HCQ 1HD0 1HDJ 1HDK 1HDM 1HDO 1HDP 1HDR 1HE7 1HF0 1HFH 1HFI 1HH8 1HI7 1HKF 1HLC 1HLG 1HLO 1HLV 1HLZ 1HML 1HP8 1HPT 1HRY 1HRZ 1HTJ 1HTN 1HTR 1HU0 1HU3 1HUC 1HUL 1HUP 1HUT 1HUW 1HW4 1HXM 1HY9 1HYI 1HYN 1HZF 1HZM 1I16 1I1J 1I1N 1I27 1I2T 1I71 1I7K 1IAM 1IAP 1IAT 1IC8 1ICH 1IFR 1IFY 1IG4 1IG6 1IHB 1IHK 1IIE 1IJB 1IJQ 1IJT 1IK9 1IKT 1ILK 1ILR 1IMH 1IMJ 1IMV 1IOJ 1IPC 1IQ3 1IQT 1IRH 1IRS 1ISI 1ITU 1ITV 1ITY 1IU1 1IUR 1IV6 1IVH 1IVY 1IXD 1IYF 1IYR 1J0W 1J1B 1J1J 1J3T 1J46 1J47 1J4W 1J5K 1J72 1J7M 1J8C 1J8E 1J8K 1J8U 1J99 1JBI 1JBQ 1JCN 1JDH 1JDP 1JDW 1JEQ 1JEY 1JFI 1JFN 1JHJ 1JI7 1JID 1JJ7 1JJR 1JKG 1JL0 1JLI 1JLJ 1JM7 1JMC 1JMQ 1JMT 1JNM 1JOC 1JPY 1JQE 1JR2 1JSF 1JSG 1JTV 1JUN 1JUO 1JV1 1JXS 1JY1 1K04 1K0M 1K1B 1K1F 1K1G 1K36 1K3Y 1K4S 1K4T 1K4U 1K5N 1K62 1K6M 1K6O 1K8F 1K8M 1K8U 1K94 1K99 1KA6 1KB2 1KB4 1KB6 1KCG 1KCQ 1KDU 1KEX 1KFU 1KGD 1KHB 1KHM 1KHX 1KI0 1KJ6 1KL9 1KMT 1KMV 1KMX 1KO6 1KO9 1KOY 1KQ6 1KQN 1KS0 1KSQ 1KT0 1KTH 1KWA 1KWM 1KXG 1KXU 1L1F 1L2H 1L2Z 1L3K 1L3Y 1L6J 1L6X 1L8R 1L9L 1L9N 1L9X 1LB6 1LCF 1LCY 1LDL 1LDR 1LE6 1LF7 1LG4 1LGP 1LI4 1LJ5 1LJM 1LKK 1LL8 1LM5 1LM7 1LMJ 1LN1 1LO1 1LO6 1LPQ 1LQV 1LSL 1LT8 1LW3 1LWV 1LWW 1LWY 1LXI 1LY2 1M1L 1M36 1M39 1M3H 1M3Q 1M48 1M54 1M5I 1M5K 1M5O 1M5P 1M5V 1M62 1M6D 1M6I 1M7K 1M7L 1M8W 1M8X 1M8Y 1M8Z 1M9I 1M9M 1M9Z 1MD8 1MEK 1MEO 1MF7 1MFG 1MFQ 1MG4 1MGX 1MH1 1MHD 1MHN 1MHW 1MIL 1MJ4 1MJN 1MKK 1MOT 1MP1 1MQ0 1MQ2 1MQ3 1MQ4 1MUZ 1MV3 1MWP 1MX3 1MZA 1N0Z 1N11 1N1F 1N1J 1N26 1N39 1N3A 1N3C 1N3L 1N45 1N5U 1N69 1N6J 1N7D 1N83 1NAF 1NB9 1NBQ 1NCN 1NCT 1ND6 1NE7 1NF1 1NFA 1NFN 1NG2 1NH3 1NHL 1NKF 1NKO 1NKP 1NKR 1NLW 1NM8 1NN5 1NNL 1NOP 1NOW 1NRG 1NRV 1NST 1NTY 1NU4 1NUB 1NUU 1NVP 1NXK 1NZI 1NZN 1NZP 1O04 1O1U 1O3X 1O4X 1O6X 1O7Z 1O8R 1OA8 1OAI 1OCT 1ODP 1OEF 1OEG 1OEH 1OEI 1OEY 1OJ6 1OK3 1OKI 1OLL 1OLZ 1ONI 1ONV 1OPP 1OQA 1OQJ 1ORF 1OTH 1OU5 1OV2 1OV3 1OW1 1OWR 1OWW 1OWX 1OXZ 1OZ2 1OZJ 1OZN 1P0R 1P0T 1P1T 1P27 1P32 1P49 1P4O 1P4U 1P57 1P5Q 1P5Z 1P6F 1P7H 1P9A 1P9O 1PB5 1PBK 1PBU 1PBW 1PC2 1PCF 1PD6 1PDG 1PE3 1PEX 1PGZ 1PJ3 1PJA 1PKX 1PL8 1PLP 1PLS 1PML 1PN5 1PO6 1POG 1POU 1PPQ 1PQN 1PR9 1PRX 1PSR 1PT6 1PV8 1PVE 1PWA 1PWB 1PZU 1Q0P 1Q1C 1Q1Q 1Q1U 1Q1V 1Q20 1Q2H 1Q2Z 1Q33 1Q38 1Q3F 1Q3X 1Q5H 1Q68 1Q7L 1Q7S 1Q8K 1Q8L 1Q8M 1Q9C 1QA9 1QB0 1QB2 1QBH 1QBJ 1QCM 1QDD 1QF8 1QG3 1QGP 1QGR 1QGV 1QH5 1QK9 1QKL 1QLZ 1QM9 1QO6 1QPC 1QQG 1QTN 1QU6 1QUB 1QUU 1QZE 1QZU 1R05 1R0D 1R1H 1R29 1R2D 1R2Q 1R3S 1R42 1R49 1R4X 1R55 1R5L 1R6J 1R6T 1R74 1R79 1RAX 1REW 1RFF 1RFI 1RG1 1RG2 1RG8 1RGO 1RGT 1RGU 1RH0 1RHF 1RHG 1RI9 1RJ8 1RJA 1RJH 1RL1 1RLW 1RMJ 1RNF 1RO4 1RR8 1RRJ 1RRK 1RTF 1RW2 1RX0 1RXD 1RXR 1RXT 1RYO 1RZ4 1RZT 1S1D 1S1E 1S1G 1S1N 1S35 1S3A 1S4W 1S79 1S7A 1S9C 1S9K 1SAW 1SBX 1SC7 1SCF 1SEN 1SEU 1SG4 1SGO 1SHU 1SI2 1SI3 1SI5 1SIQ 1SJ6 1SJF 1SJQ 1SJR 1SK4 1SL6 1SLM 1SMD 1SMO 1SNL 1SNZ 1SO2 1SO7 1SOH 1SPJ 1SQW 1SRA 1SRQ 1SRS 1SS6 1SSL 1SSP 1ST0 1SU3 1SVC 1SXE 1SZ7 1SZB 1T0C 1T15 1T2A 1T2J 1T2K 1T38 1T39 1T3G 1T3N 1T3Y 1T5C 1T5I 1T6N 1T77 1T7H 1T7V 1T84 1T8I 1T8T 1T91 1T94 1TAZ 1TBF 1TDH 1TEN 1TEV 1TFF 1TFI 1TG6 1TGH 1TGR 1TH0 1TKI 1TKN 1TL8 1TMR 1TNR 1TOZ 1TP4 1TPG 1TPM 1TR2 1TSR 1TTN 1TTX 1TUP 1TUZ 1TV9 1TVA 1TVD 1TVG 1TVX 1TXD 1TXP 1U1K 1U1L 1U1M 1U1N 1U1O 1U1P 1U1Q 1U1R 1U2H 1U37 1U39 1U3B 1U3W 1U5D 1U5M 1U5S 1U6B 1U6T 1U7B 1UA2 1UAP 1UBD 1UC6 1UCT 1UCV 1UDL 1UE9 1UEL 1UEM 1UEN 1UEP 1UEQ 1UEW 1UEY 1UF0 1UF1 1UFF 1UFI 1UFW 1UFX 1UG1 1UG3 1UGK 1UHC 1UHP 1UII 1UIT 1UJ2 1UJD 1UJS 1UJT 1UJU 1UJV 1UM1 1UM7 1UMK 1UNC 1UNQ 1UOH 1UOU 1UPK 1UPQ 1URN 1US0 1USE 1UUC 1UUH 1UV0 1UVF 1UVG 1UVQ 1UW0 1UW4 1UW5 1UWY 1UX6 1UYA 1UYB 1UZ3 1UZC 1UZE 1UZK 1V05 1V1C 1V27 1V5J 1V5W 1V62 1V66 1V6G 1V84 1V88 1V89 1V95 1V9V 1VA1 1VA2 1VA3 1VA9 1VBX 1VBY 1VBZ 1VC0 1VC5 1VC6 1VC7 1VCA 1VD4 1VEC 1VFC 1VIG 1VKA 1VRY 1VTN 1VYB 1VZJ 1VZO 1W0H 1W0R 1W0T 1W0U 1W1H 1W24 1W2F 1W3B 1W4M 1W4R 1W4V 1W6K 1W6U 1W6V 1W70 1W9C 1W9E 1WAK 1WAO 1WAR 1WB0 1WBR 1WCH 1WD2 1WDY 1WEL 1WER 1WEZ 1WF0 1WF1 1WF5 1WF6 1WF8 1WFM 1WFN 1WFO 1WFQ 1WG1 1WG5 1WG7 1WGD 1WGL 1WGM 1WGN 1WGO 1WGR 1WGV 1WGX 1WGY 1WH0 1WH1 1WH3 1WH9 1WHA 1WHB 1WHL 1WHM 1WHR 1WI1 1WI3 1WI5 1WI8 1WIE 1WIG 1WIL 1WIM 1WIO 1WIS 1WIZ 1WJ3 1WJ4 1WJ6 1WJI 1WJM 1WJP 1WJQ 1WJR 1WJS 1WJU 1WK0 1WKU 1WL4 1WLJ 1WLX 1WLZ 1WM3 1WMA 1WMH 1WMS 1WMV 1WOJ 1WOU 1WPA 1WQ6 1WQJ 1WRM 1WSR 1WT6 1WTB 1WU9 1WUU 1WVN 1WVO 1WWB 1WWC 1WWT 1WWU 1WWW 1WWX 1WWY 1WX6 1WX7 1WXM 1WXP 1WXS 1WXV 1WY8 1WYM 1WYN 1WYO 1WYQ 1WYR 1WYX 1WZ0 1WZ9 1WZV 1X0F 1X0H 1X0V 1X1F 1X1G 1X2L 1X2P 1X2Q 1X3A 1X3C 1X3D 1X3H 1X3S 1X40 1X41 1X44 1X47 1X4A 1X4B 1X4E 1X4G 1X4I 1X4J 1X4P 1X4Q 1X4S 1X4U 1X4V 1X4W 1X4X 1X50 1X51 1X52 1X53 1X57 1X59 1X5C 1X5D 1X5E 1X5H 1X5M 1X5N 1X5O 1X5P 1X5Q 1X5R 1X5T 1X5U 1X5W 1X5X 1X5Z 1X61 1X62 1X63 1X65 1X66 1X67 1X68 1X6A 1X6B 1X6D 1X6F 1X6G 1X6H 1X6V 1X79 1X8B 1X8Y 1X9D 1X9N 1X9Q 1XAW 1XC5 1XCR 1XD4 1XED 1XFD 1XFE 1XG1 1XG5 1XGW 1XHN 1XJD 1XJV 1XK5 1XK8 1XKE 1XKI 1XKS 1XM9 1XMK 1XO5 1XPH 1XQR 1XR0 1XSL 1XSN 1XSP 1XTI 1XU9 1XW3 1XWE 1XWI 1XX0 1Y02 1Y2K 1Y3J 1Y4E 1Y4J 1Y4M 1Y7N 1Y7Q 1Y7X 1Y8Q 1Y8X 1Y93 1Y96 1Y97 1YA0 1YB1 1YB5 1YC0 1YCK 1YDE 1YDL 1YFH 1YGR 1YGS 1YH2 1YIB 1YJR 1YNW 1YO5 1YO8 1YOV 1YPQ 1YQB 1YQK 1YQL 1YQM 1YQR 1YRK 1YRV 1YSE 1YSX 1YTY 1YUK 1YVL 1YW9 1YWI 1YX4 1YXM 1YYB 1YYH 1YZ1 1YZA 1YZM 1Z08 1Z4R 1Z60 1Z6C 1Z6T 1Z6U 1Z6V 1Z70 1Z8G 1Z9E 1Z9I 1Z9M 1Z9Q 1ZA4 1ZAQ 1ZBH 1ZBQ 1ZBU 1ZCM 1ZD1 1ZD8 1ZD9 1ZDN 1ZET 1ZGK 1ZH5 1ZIW 1ZJK 1ZJM 1ZJN 1ZKC 1ZKH 1ZKL 1ZLG 1ZLM 1ZMD 1ZMI 1ZMM 1ZMZ 1ZN8 1ZOQ 1ZQ9 1ZQA 1ZQB 1ZQC 1ZQD 1ZQE 1ZQF 1ZQG 1ZQH 1ZQI 1ZQJ 1ZQK 1ZQL 1ZQM 1ZQN 1ZQO 1ZQP 1ZQQ 1ZQR 1ZQS 1ZQT 1ZR3 1ZR9 1ZRH 1ZS6 1ZS9 1ZSQ 1ZSX 1ZSY 1ZT3 1ZTG 1ZTO 1ZUO 1ZV4 1ZXA 1ZXM 1ZXQ 1ZY7 1ZZA 1ZZI 1ZZJ 1ZZK 1ZZN 1ZZP 1ZZW 2A01 2A07 2A14 2A1H 2A1I 2A1J 2A1R 2A1S 2A1X 2A25 2A26 2A2C 2A2K 2A2N 2A2R 2A38 2A3J 2A4D 2A55 2A5P 2A5R 2A66 2A72 2A7L 2A7O 2A8J 2A91 2A9U 2ABL 2AC0 2AC3 2ACJ 2ACM 2ACX 2AD9 2ADB 2ADC 2ADY 2AEB 2AEX 2AFF 2AG4 2AG5 2AHI 2AHX 2AKZ 2ALD 2ALZ 2ANN 2ANR 2ANY 2AQ0 2AR5 2ARR 2ARW 2AS5 2ASK 2ASU 2ATA 2ATV 2AUG 2AVD 2AW2 2AXI 2AXL 2AXN 2AXY 2AYN 2AZE 2AZX 2B0O 2B1W 2B25 2B2Y 2B3G 2B3H 2B3Y 2B49 2B5N 2B69 2B6F 2B86 2B8W 2B9E 2BB5 2BBA 2BBS 2BBY 2BCQ 2BCR 2BCS 2BCU 2BCV 2BDG 2BEC 2BFD 2BH9 2BID 2BJ6 2BJN 2BJX 2BK8 2BKA 2BKF 2BO9 2BOU 2BP1 2BP4 2BRF 2BSK 2BTA 2BTB 2BUG 2BUJ 2BW0 2BYE 2BZ6 2BZF 2BZG 2BZL 2BZY 2C0C 2C2N 2C35 2C3N 2C43 2C46 2C4J 2C60 2C62 2C6A 2C6Q 2C6U 2C6Y 2C7A 2C7H 2C7W 2C95 2C9A 2C9O 2C9Y 2CAR 2CB5 2CB8 2CBZ 2CCQ 2CE2 2CEF 2CEH 2CEO 2CEZ 2CFJ 2CG7 2CH0 2CH5 2CH9 2CIH 2CKA 2CKC 2CKK 2CL3 2CLQ 2CLT 2CO8 2COB 2COC 2COD 2COE 2COF 2COK 2COM 2COO 2COP 2COR 2COT 2COY 2COZ 2CP5 2CP6 2CP8 2CP9 2CPC 2CPE 2CPM 2CPN 2CPQ 2CPR 2CPT 2CPW 2CPX 2CPY 2CQ0 2CQ1 2CQ2 2CQ3 2CQ4 2CQ7 2CQ8 2CQA 2CQB 2CQC 2CQD 2CQE 2CQF 2CQG 2CQH 2CQI 2CQJ 2CQK 2CQL 2CQM 2CQN 2CQO 2CQQ 2CQR 2CQU 2CQV 2CQY 2CR3 2CR4 2CR6 2CR8 2CR9 2CRC 2CRE 2CRF 2CRM 2CRR 2CRU 2CRW 2CRY 2CRZ 2CS0 2CS1 2CS2 2CS3 2CS4 2CS5 2CS8 2CSF 2CSH 2CSK 2CSO 2CSP 2CSQ 2CSS 2CSV 2CSW 2CSY 2CSZ 2CT0 2CT1 2CT2 2CT4 2CT5 2CT6 2CT7 2CTD 2CTE 2CTF 2CTJ 2CTK 2CTL 2CTM 2CTO 2CTQ 2CTT 2CTU 2CU1 2CU7 2CU8 2CUB 2CUD 2CUF 2CUH 2CUI 2CUM 2CUP 2CV5 2CVD 2CW6 2CW9 2CXK 2CXY 2D0T 2D2P 2D39 2D46 2D48 2D4C 2D58 2D5R 2D68 2D7I 2D7L 2D85 2D86 2D87 2D89 2D8H 2D8I 2D8K 2D8M 2D8R 2D8S 2D8T 2D8U 2D8X 2D8Y 2D8Z 2D92 2D93 2D96 2D99 2D9C 2D9D 2D9F 2D9G 2D9H 2D9I 2D9K 2D9L 2D9M 2D9N 2D9O 2D9P 2D9U 2D9W 2D9X 2D9Z 2DA0 2DA3 2DA4 2DA6 2DA7 2DAD 2DAE 2DAF 2DAG 2DAH 2DAI 2DAJ 2DAK 2DAL 2DAM 2DAO 2DAQ 2DAR 2DAS 2DAT 2DAV 2DAW 2DAX 2DAZ 2DB2 2DB5 2DB6 2DB7 2DB8 2DBA 2DBD 2DBF 2DBG 2DBH 2DBJ 2DBK 2DC2 2DC3 2DCE 2DCO 2DCR 2DDF 2DDI 2DE0 2DFD 2DGP 2DGR 2DGS 2DGW 2DGX 2DGY 2DGZ 2DH2 2DH7 2DH8 2DH9 2DHA 2DHG 2DHJ 2DHK 2DHO 2DHS 2DHX 2DHY 2DHZ 2DI0 2DI7 2DIA 2DIB 2DID 2DIG 2DII 2DIM 2DIN 2DIP 2DIQ 2DIR 2DIS 2DIT 2DIU 2DIV 2DIW 2DIX 2DIY 2DJ0 2DJ7 2DJ8 2DJA 2DJB 2DJF 2DJP 2DJR 2DJS 2DJT 2DJU 2DJV 2DK1 2DK2 2DK3 2DK4 2DK6 2DK7 2DK9 2DKL 2DKM 2DKO 2DKR 2DKS 2DKU 2DKX 2DKY 2DKZ 2DL0 2DL1 2DL4 2DL5 2DL6 2DL8 2DL9 2DLE 2DLH 2DLK 2DLL 2DLO 2DLP 2DLS 2DLU 2DLW 2DLX 2DLZ 2DM2 2DM3 2DM4 2DM8 2DMB 2DMC 2DMD 2DME 2DMF 2DMG 2DMH 2DMI 2DMJ 2DMK 2DMM 2DMN 2DMO 2DMQ 2DMW 2DMY 2DMZ 2DN0 2DN6 2DN7 2DN8 2DNC 2DNE 2DNF 2DNH 2DNL 2DNM 2DNN 2DNP 2DNR 2DNT 2DNW 2DNX 2DNZ 2DO0 2DO1 2DO3 2DO4 2DO5 2DO7 2DOC 2DOD 2DOE 2DOF 2DOK 2DPI 2DPJ 2DQ5 2DS4 2DSC 2DT6 2DT7 2DUN 2DVJ 2DW4 2DWY 2DYB 2DYL 2DYQ 2DZI 2DZJ 2DZL 2DZM 2E0A 2E0J 2E0T 2E19 2E1F 2E1O 2E1Q 2E29 2E2R 2E2W 2E3H 2E3I 2E3L 2E3N 2E3V 2E42 2E43 2E44 2E45 2E50 2E56 2E5G 2E5H 2E5J 2E5K 2E5N 2E5O 2E5R 2E5S 2E5Z 2E60 2E63 2E6I 2E6J 2E6N 2E6O 2E6P 2E6Q 2E6R 2E6S 2E6W 2E6Z 2E70 2E71 2E72 2E73 2E7A 2E7C 2E7G 2E7H 2E7K 2E7M 2E7O 2E8D 2E8M 2E8N 2E8O 2E8P 2E9G 2E9H 2E9I 2E9K 2E9L 2E9X 2EA5 2EA6 2EAM 2EAN 2EAO 2EAP 2EAQ 2EB1 2EBK 2EBM 2EBP 2EBQ 2EBR 2EBT 2EBU 2EBV 2EBW 2EBZ 2EC1 2EC3 2EC4 2EC8 2ECB 2ECC 2ECI 2ECJ 2ECL 2ECN 2ECY 2ED1 2ED2 2ED7 2ED8 2ED9 2EDB 2EDD 2EDE 2EDF 2EDJ 2EDK 2EDN 2EDO 2EDP 2EDU 2EDV 2EDX 2EDY 2EE0 2EE1 2EE2 2EE3 2EE4 2EE7 2EE9 2EEA 2EEB 2EEC 2EEF 2EEH 2EEI 2EEL 2EFI 2EFK 2EGA 2EGC 2EGD 2EGE 2EGM 2EGP 2EGQ 2EH0 2EHE 2EHF 2EHR 2EJ4 2EJ7 2EJ8 2EJE 2EJM 2EJY 2EK1 2EKF 2EKH 2EKI 2EKJ 2EKK 2EKO 2EKX 2EL8 2ELA 2ELI 2ELM 2ELN 2ELO 2ELP 2ELU 2ELV 2ENK 2ENN 2ENO 2ENP 2ENQ 2ENV 2ENY 2ENZ 2EO1 2EO3 2EO9 2EOC 2EOD 2EP4 2EP6 2EP8 2EPA 2EPB 2EPD 2EPP 2EQE 2EQF 2EQG 2EQK 2EQM 2EQN 2EQO 2EQR 2EQS 2EQU 2EQX 2EQZ 2ERF 2ERR 2ESK 2ETT 2EU9 2EVA 2EVZ 2EW9 2EX4 2EXG 2EYV 2EYW 2EYY 2EZD 2EZE 2EZF 2EZG 2F15 2F1Z 2F37 2F3I 2F4W 2F5J 2F5K 2F5Y 2F60 2F69 2F6Q 2F71 2F73 2F83 2F8A 2F8X 2F8Y 2F9L 2FAU 2FAZ 2FBE 2FBM 2FBY 2FC6 2FC7 2FC8 2FC9 2FCF 2FCW 2FDV 2FE5 2FEB 2FFQ 2FFU 2FFW 2FG5 2FH1 2FH7 2FHO 2FJ4 2FK9 2FKL 2FLL 2FLN 2FLP 2FLY 2FMA 2FMP 2FMQ 2FMR 2FMS 2FN2 2FNB 2FO0 2FOZ 2FRG 2FRY 2FUE 2FUU 2FV2 2FV7 2FVV 2FXM 2FY1 2FY2 2FY7 2FZP 2G1L 2G2K 2G30 2G3R 2G4B 2G4C 2G62 2G6Z 2G76 2G7B 2G7R 2GA7 2GAO 2GCG 2GD5 2GDZ 2GEE 2GF0 2GF5 2GF9 2GFO 2GFU 2GGM 2GGT 2GGZ 2GHF 2GHT 2GI7 2GKU 2GLI 2GMF 2GOW 2GQI 2GQJ 2GRA 2GRC 2GRY 2GSB 2GSX 2GTG 2GTJ 2GTR 2GUT 2GW6 2GWS 2GXB 2GY5 2GYS 2GYT 2GYZ 2GZV 2H00 2H0D 2H2B 2H2M 2H2T 2H31 2H3L 2H3N 2H41 2H4U 2H4V 2H57 2H58 2H5G 2H63 2H6D 2H6F 2H7C 2H7T 2H8H 2H8L 2H8N 2H8R 2HA1 2HA8 2HAC 2HAZ 2HC1 2HCC 2HDL 2HDZ 2HE4 2HEH 2HF5 2HF6 2HGL 2HGN 2HGS 2HH2 2HH3 2HHJ 2HI4 2HJ8 2HKY 2HLW 2HM2 2HO2 2HP4 2HQH 2HQQ 2HQX 2HR0 2HR7 2HST 2HT9 2HTF 2HVZ 2HW4 2HW5 2HWY 2HXP 2HXS 2HYI 2HYN 2HYV 2HZ5 2HZ6 2HZC 2HZD 2HZQ 2I1Y 2I32 2I3B 2I3H 2I46 2I4I 2I4K 2I50 2I53 2I5F 2I5O 2I5W 2I6L 2I6T 2I75 2I7A 2I7D 2I7K 2I7V 2I99 2I9A 2I9G 2I9P 2IB8 2IBN 2ICC 2ID5 2IDX 2IF1 2IF5 2IF7 2IGP 2IHD 2II0 2IIK 2IIM 2IJA 2ILA 2ILR 2IMS 2IPX 2IQ1 2IQC 2IQJ 2ISO 2ISP 2IU1 2IUH 2IUW 2IV4 2IV5 2IVX 2IW2 2IWL 2IWN 2IWQ 2IWR 2IWZ 2IYB 2IYK 2IZR 2IZX 2IZZ 2J05 2J0I 2J0Q 2J0S 2J1D 2J2S 2J32 2J3S 2J5D 2J67 2J6F 2J6L 2J76 2J7T 2J8B 2J8H 2J8J 2J8P 2J8Z 2J91 2J9L 2JA4 2JAK 2JAM 2JBH 2JBM 2JC9 2JE0 2JEO 2JFK 2JGB 2JGN 2JGW 2JI4 2JIF 2JII 2JIK 2JIL 2JIS 2JJD 2JJU 2JK2 2JK4 2JKU 2JLL 2JLP 2JM4 2JMD 2JMO 2JNB 2JNH 2JO1 2JOA 2JOD 2JOX 2JP0 2JP1 2JP2 2JP9 2JPA 2JPD 2JQ3 2JQ6 2JQ8 2JR7 2JRF 2JRH 2JRJ 2JRS 2JRZ 2JS2 2JSN 2JTF 2JTG 2JTX 2JUF 2JUJ 2JUN 2JV5 2JVN 2JVZ 2JW2 2JW4 2JW5 2JW6 2JWX 2JX2 2JX3 2JX8 2JXB 2JXD 2JXJ 2JXW 2JXY 2JY5 2JYI 2JYT 2JZX 2K18 2K1B 2K1L 2K1M 2K1P 2K21 2K27 2K2C 2K2D 2K2I 2K2M 2K2O 2K3G 2K3W 2K40 2K6B 2K6G 2K6M 2K6O 2K6S 2K7B 2K7C 2K7F 2K7N 2K7P 2K7Q 2K85 2K86 2K89 2K8G 2K8O 2K8P 2K9A 2K9G 2K9U 2K9Y 2KA1 2KA3 2KAP 2KAV 2KBG 2KBI 2KBS 2KCC 2KCX 2KDB 2KDD 2KDG 2KDK 2KDP 2KE1 2KE4 2KE7 2KE9 2KEA 2KEB 2KEO 2KES 2KFV 2KFY 2KG0 2KG1 2KG5 2KGR 2KGT 2KHX 2KIE 2KIJ 2KIQ 2KIS 2KIU 2KIV 2KIZ 2KJM 2KJX 2KJY 2KK0 2KK1 2KK6 2KKF 2KKQ 2KKR 2KKT 2KKW 2KL7 2KLD 2KLL 2KLU 2KLZ 2KM6 2KMS 2KMU 2KMV 2KMZ 2KN6 2KN7 2KN8 2KNA 2KNC 2KNH 2KNO 2KNV 2KNX 2KNY 2KO0 2KOE 2KOM 2KOY 2KPE 2KPF 2KPK 2KQB 2KQP 2KR0 2KR1 2KR6 2KRB 2KRG 2KRK 2KRR 2KS1 2KS9 2KSN 2KSP 2KSR 2KT0 2KTU 2KU3 2KU7 2KUM 2KUO 2KUP 2KV2 2KV3 2KV8 2KVE 2KVR 2KW1 2KW3 2KW6 2KW9 2KWH 2KX8 2KXJ 2KXN 2KXQ 2KXR 2KY5 2KYG 2KYK 2KYU 2KZ3 2KZA 2KZU 2L03 2L08 2L0A 2L0B 2L0E 2L1C 2L1G 2L1I 2L1J 2L1L 2L1Q 2L1X 2L27 2L2D 2L2J 2L2L 2L2O 2L30 2L31 2L33 2L34 2L3D 2L3G 2L3L 2L3X 2L4C 2L4M 2L4N 2L54 2L5C 2L5D 2L5F 2L5G 2L5I 2L5U 2L5V 2L63 2L6A 2L6K 2L6L 2L6U 2L6W 2L73 2L75 2L76 2L77 2L7B 2L7M 2L7R 2L7S 2L7T 2L7Z 2L80 2L81 2L87 2L8E 2L8S 2L91 2L98 2L9I 2L9M 2L9N 2L9R 2L9U 2L9Z 2LA5 2LA6 2LAJ 2LAT 2LAU 2LBC 2LBF 2LBG 2LC3 2LCC 2LCD 2LCE 2LCM 2LCW 2LCX 2LD0 2LD2 2LD4 2LDM 2LDU 2LDY 2LE3 2LE7 2LE8 2LEA 2LEB 2LEC 2LEH 2LEO 2LFE 2LFG 2LFH 2LG1 2LGP 2LGQ 2LGW 2LGX 2LGY 2LHA 2LI8 2LJ0 2LJD 2LJK 2LK0 2LK2 2LK9 2LKJ 2LKN 2LKO 2LKQ 2LKS 2LKX 2LKZ 2LL2 2LLH 2LLK 2LLP 2LLX 2LLY 2LM0 2LM5 2LMB 2LMD 2LMF 2LMG 2LMI 2LMJ 2LMR 2LNA 2LNB 2LNE 2LNF 2LNG 2LNI 2LNL 2LNW 2LO1 2LO4 2LOB 2LOH 2LOM 2LON 2LOO 2LOQ 2LOR 2LOT 2LP1 2LQ6 2LQL 2LQT 2LQW 2LR8 2LRI 2LRR 2LS2 2LS3 2LS4 2LS8 2LSO 2LSQ 2LSR 2LSW 2LT7 2LTM 2LTP 2LTU 2LTV 2LU7 2LUB 2LUL 2LUV 2LV2 2LV7 2LV9 2LVA 2LVC 2LVN 2LVR 2LVT 2LVU 2LW4 2LW9 2LWD 2LX7 2LXI 2LXL 2LXS 2LXU 2LY4 2LY9 2LYH 2LYW 2LZ1 2M09 2M0C 2M0D 2M0E 2M0F 2M0O 2M0P 2M0R 2M0T 2M0V 2M13 2M17 2M1L 2M20 2M2B 2M2E 2M2F 2M34 2M38 2M3D 2M5O 2M5V 2M6N 2M6Y 2M7S 2M9I 2MHU 2NLK 2NLL 2NLS 2NLW 2NML 2NMS 2NN6 2NNT 2NNY 2NO2 2NOB 2NOE 2NOF 2NOH 2NOI 2NOL 2NOZ 2NPL 2NPT 2NPU 2NQ3 2NQC 2NR1 2NSM 2NT0 2NT2 2NTE 2NW2 2NWM 2NXP 2NYT 2NYU 2NZ2 2NZ4 2NZ6 2NZ7 2NZI 2NZL 2O07 2O10 2O13 2O23 2O28 2O2K 2O2O 2O2T 2O36 2O3H 2O3M 2O49 2O4A 2O4X 2O61 2O6G 2O6L 2O71 2O72 2O8B 2O8C 2O8D 2O8E 2O8F 2O93 2O95 2O9S 2OAT 2OAY 2OB0 2OBD 2OBI 2OC3 2OCF 2OCG 2OCP 2OCT 2OD1 2ODC 2ODD 2ODP 2ODV 2OEH 2OEX 2OH2 2OHF 2OIB 2OIH 2OIT 2OJ2 2OJ3 2OJW 2OK3 2OKV 2OLM 2OM5 2OO0 2OO9 2OOA 2OOQ 2OP7 2OPG 2OPU 2OPV 2OPW 2OQ0 2OQ1 2OQ5 2ORV 2OS6 2OSA 2OU1 2OUC 2OUD 2OUS 2OVC 2OVJ 2OWI 2OX8 2OXC 2OXM 2OYC 2OYT 2OZB 2P01 2P02 2P0A 2P0D 2P0K 2P0W 2P1B 2P1T 2P23 2P26 2P2R 2P39 2P3W 2P4K 2P57 2P5S 2P5X 2P64 2P66 2P6N 2P6V 2P6X 2P8E 2P8V 2P9R 2PA1 2PA2 2PB7 2PBC 2PD6 2PE4 2PE8 2PET 2PEZ 2PF5 2PFI 2PFN 2PFO 2PFP 2PFQ 2PI0 2PI2 2PID 2PIE 2PKD 2PL3 2PMV 2PMY 2PN8 2PNT 2POI 2POM 2PPI 2PPL 2OXE 2PPN 2PQ5 2PQ8 2PQF 2PQU 2PRT 2PSO 2PSQ 2PUY 2PV0 2PXI 2PXX 2PY9 2PZ1 2PZD 2PZE 2Q0Z 2Q12 2Q13 2Q20 2Q2F 2Q3E 2Q3G 2Q3Z 2Q4K 2Q4Q 2Q4V 2Q51 2Q5X 2Q7D 2Q7M 2Q7Z 2Q80 2Q81 2Q87 2Q8K 2Q8R 2Q8T 2Q9V 2QAG 2QBW 2QC7 2QCQ 2QDJ 2QFA 2QFD 2QFE 2QFG 2QFH 2QFJ 2QFZ 2QG1 2QGX 2QIS 2QJ2 2QJF 2QJZ 2QK4 2QK9 2QKB 2QKK 2QKQ 2QM4 2QNA 2QND 2QNK 2QNR 2QOL 2QPW 2QQ2 2QQ5 2QQ8 2QQH 2QQI 2QQM 2QRV 2QS9 2QSQ 2QT1 2QTW 2QTZ 2QXI 2QY0 2QY7 2QYP 2QYQ 2QZ4 2QZD 2QZF 2QZH 2R0B 2R15 2R2J 2R2N 2R2O 2R37 2R3A 2R3V 2R4F 2R55 2R5T 2R83 2R8U 2RA4 2RB4 2RB8 2RBA 2RCT 2RCZ 2RE9 2REI 2REP 2REY 2RF0 2RG8 2RGF 2RGZ 2RI7 2RIE 2RIQ 2RJQ 2RK3 2RKB 2RKU 2RKY 2RLO 2RLP 2RLQ 2RMJ 2RMX 2RND 2RNL 2RNQ 2RO1 2ROP 2ROR 2RPC 2RPJ 2RPP 2RPR 2RQ1 2RQ4 2RQC 2RQP 2RQT 2RR4 2RR6 2RR8 2RRA 2RRB 2RRD 2RRF 2RRI 2RRS 2RS9 2RSG 2RSH 2RSI 2RSJ 2RSQ 2SHP 2SSP 2STT 2STW 2TGI 2TMP 2U1A 2U2F 2UP1 2UUR 2UV4 2UWN 2UWQ 2UX0 2UXW 2UYY 2UZ8 2UZG 2UZK 2V0E 2V0F 2V0O 2V14 2V1N 2V1X 2V37 2V3Q 2V3S 2V40 2V4B 2V4U 2V5F 2V5N 2V5O 2V5T 2V5Y 2V62 2V66 2V6Z 2V70 2V76 2V7R 2V9K 2V9R 2V9T 2V9Y 2VAC 2VAJ 2VC8 2VDX 2VE7 2VFK 2VFX 2VGE 2VH7 2VHF 2VIF 2VIG 2VJ2 2VJ3 2VJE 2VKC 2VKP 2VKQ 2VKW 2VM5 2VN8 2VNF 2VO1 2VOD 2VON 2VOO 2VOP 2VPB 2VPH 2VPI 2VPJ 2VPK 2VQ3 2VQM 2VRD 2VRE 2VRG 2VSP 2VSV 2VSW 2VSZ 2VT8 2VUQ 2VUW 2VVK 2VWR 2VX2 2VX3 2VXD 2VXO 2VXP 2VY5 2VYI 2VZ5 2VZC 2W0G 2W0I 2W0P 2W0T 2W18 2W2J 2W2N 2W3C 2W4F 2W4L 2W4O 2W4R 2W50 2W51 2W5A 2W5Y 2W7A 2W7O 2W7P 2W84 2W86 2W8N 2WA0 2WA7 2WAX 2WBI 2WCE 2WCY 2WEF 2WFD 2WFH 2WFI 2WGH 2WGP 2WH5 2WIM 2WJW 2WJY 2WL1 2WL8 2WM1 2WM3 2WM8 2WNG 2WNO 2WNP 2WNS 2WNT 2WO1 2WPH 2WQ9 2WQI 2WQJ 2WQM 2WQR 2WUL 2WVI 2WVR 2WWE 2WWW 2WWY 2WX3 2WXW 2WYA 2WYQ 2WZ1 2WZ9 2WZB 2WZO 2X1W 2X29 2X2E 2X2U 2X36 2X4D 2X4F 2X5Y 2X6U 2X7A 2X7F 2X8A 2XA6 2XB1 2XB2 2XC7 2XDG 2XDP 2XDV 2XEB 2XES 2XEU 2XFN 2XHI 2XIJ 2XIO 2XJY 2XMR 2XN4 2XN6 2XOC 2XR6 2XRC 2XRI 2XRW 2XS6 2XSQ 2XSS 2XST 2XSW 2XSZ 2XTC 2XTD 2XTP 2XU3 2XU7 2XUB 2XUS 2XV5 2XVS 2XVT 2XW9 2XXZ 2XY1 2XY2 2XYC 2XZE 2XZG 2XZP 2XZZ 2Y05 2Y1H 2Y1N 2Y1Y 2Y23 2Y25 2Y29 2Y2A 2Y3J 2Y3K 2Y3L 2Y43 2Y4Q 2Y4T 2Y5C 2Y6E 2Y73 2Y7B 2Y7J 2Y8G 2Y95 2Y96 2Y9A 2Y9B 2Y9C 2Y9D 2Y9U 2YAD 2YAN 2YB6 2YBX 2YCF 2YD0 2YD6 2YD9 2YDL 2YDY 2YEX 2YF0 2YG2 2YGD 2YGN 2YGO 2YGQ 2YGW 2YH0 2YH1 2YHF 2YHW 2YKG 2YKO 2YLE 2YLM 2YMB 2YMK 2YPD 2YPR 2YPS 2YQG 2YQI 2YQK 2YQL 2YQP 2YQQ 2YQR 2YR3 2YRA 2YRB 2YRC 2YRE 2YRG 2YRL 2YRN 2YRO 2YRP 2YRQ 2YRT 2YRV 2YRY 2YRZ 2YS0 2YS2 2YS4 2YS8 2YS9 2YSA 2YSC 2YSH 2YSJ 2YSL 2YSM 2YSQ 2YSR 2YST 2YSX 2YT4 2YT7 2YT8 2YT9 2YTC 2YTU 2YTV 2YTW 2YTX 2YTY 2YU1 2YU3 2YU4 2YU6 2YU8 2YUA 2YUC 2YUD 2YUF 2YUH 2YUJ 2YUK 2YUM 2YUN 2YUQ 2YUR 2YUS 2YUU 2YUW 2YUX 2YUY 2YUZ 2YVI 2YVQ 2YVR 2YW8 2YWK 2YX8 2YXM 2YXT 2YY0 2YYN 2YYO 2YZ8 2Z0B 2Z0U 2Z0W 2Z14 2Z15 2Z17 2Z3Q 2Z5D 2Z5E 2Z5K 2Z6E 2Z6H 2Z6O 2ZAJ 2ZB4 2ZEJ 2ZFH 2ZFU 2ZFY 2ZG1 2ZGC 2ZHN 2ZJ3 2ZKM 2ZMD 2ZMF 2ZND 2ZNR 2ZOU 2ZQQ 2ZT5 2ZV2 2ZV6 2ZW3 3A03 3A1A 3A1B 3A1F 3A1J 3A2A 3A4U 3A6N 3A77 3A7I 3A98 3A99 3AAF 3ABD 3ABH 3ADL 3AFA 3AGV 3AGY 3AHQ 3AIH 3AJ4 3AKM 3AL2 3AL5 3AN2 3AOX 3AP1 3AP9 3APA 3AQG 3AQI 3AQQ 3ASK 3ASL 3AU4 3AV1 3AV2 3AVR 3AY5 3AYW 3AZE 3AZF 3AZG 3AZH 3AZI 3AZJ 3AZK 3AZL 3AZM 3AZN 3B0T 3B2D 3B5H 3B6E 3B6H 3B6R 3B6U 3B7K 3B7X 3B7Y 3B83 3B84 3B8K 3B93 3B95 3B9C 3BBB 3BCH 3BCZ 3BDL 3BE8 3BEJ 3BER 3BFN 3BFO 3BG9 3BGS 3BGV 3BHD 3BHY 3BI7 3BIY 3BJ4 3BJ5 3BJ9 3BJC 3BJU 3BKB 3BL9 3BO2 3BO3 3BO4 3BO5 3BOR 3BPJ 3BPT 3BPU 3BQ7 3BQO 3BQP 3BRB 3BRV 3BS9 3BSB 3BSU 3BSX 3BTB 3BTX 3BTY 3BTZ 3BU0 3BU8 3BUC 3BUX 3BVO 3BWY 3BXW 3BYI 3BZH 3C0I 3C1V 3C1X 3C2I 3C2K 3C2L 3C2M 3C3R 3C5C 3C5E 3C5F 3C5G 3C5H 3C5K 3C5N 3C5R 3C5V 3C6K 3C6W 3C7X 3C8X 3C9Q 3CAF 3CB2 3CBB 3CBZ 3CEG 3CEK 3CH4 3CI9 3CJJ 3CJW 3CKK 3CLZ 3CMY 3CO6 3COA 3COG 3COK 3COO 3CPF 3CQC 3CQV 3CRD 3CRY 3CTR 3CTZ 3CU7 3CUL 3CUN 3CUQ 3CVF 3CW1 3CW3 3CWW 3CX2 3CXL 3CYY 3CZH 3D06 3D0A 3D1N 3D2N 3D2Q 3D2S 3D32 3D34 3D3J 3D3K 3D3L 3D3M 3D4J 3D59 3D6M 3D7C 3D8B 3D8D 3D9H 3D9N 3D9S 3D9T 3DAD 3DAI 3DAK 3DAL 3DB3 3DB5 3DCY 3DD2 3DDS 3DDT 3DDU 3DEM 3DH1 3DJ9 3DK9 3DKM 3DKP 3DLJ 3DLM 3DLQ 3DLS 3DLX 3DPL 3DRX 3DRZ 3DSH 3DTC 3DWB 3DWD 3DXE 3DXT 3DYD 3DYN 3DYT 3DZU 3DZY 3E00 3E04 3E0G 3E0J 3E1I 3E1R 3E21 3E3R 3E46 3E4C 3E6U 3E77 3E7E 3E9K 3E9L 3E9V 3EAB 3EAP 3EAY 3EAZ 3EBB 3EBQ 3EC8 3ECM 3ECR 3ECS 3ED7 3EDH 3EDU 3EDV 3EDY 3EG3 3EGA 3EGI 3EGN 3EGZ 3EH1 3EH2 3EHR 3EHW 3EIJ 3EJH 3ELB 3ELO 3EMW 3ENP 3EO2 3EOP 3EP0 3EPG 3EPI 3EPZ 3EQ5 3EQC 3EQT 3ERB 3ERY 3ETO 3EU9 3EVI 3EW8 3EWS 3EWY 3EX7 3EXE 3EY6 3EYI 3EZQ 3F02 3F04 3F0W 3F1I 3F1P 3F1R 3F21 3F22 3F23 3F2K 3F31 3F3S 3F4M 3F59 3F5O 3F66 3F6K 3F6Q 3F6U 3F6Y 3F70 3F7Q 3F81 3F8U 3F9M 3F9X 3FAU 3FAY 3FB2 3FBK 3FBY 3FCI 3FCX 3FD5 3FDO 3FDR 3FDW 3FE2 3FE3 3FE4 3FEA 3FED 3FEG 3FFL 3FFM 3FFN 3FG7 3FGH 3FHR 3FHT 3FIA 3FIB 3FK2 3FKC 3FL2 3FL7 3FLG 3FLV 3FM0 3FME 3FMZ 3FO5 3FRR 3FRT 3FRV 3FS1 3FSO 3FVO 3FVS 3FVY 3FW3 3FX0 3FXT 3FY1 3G07 3G0H 3G2F 3G2S 3G36 3G4E 3G4G 3G5C 3G5P 3G6V 3G6X 3G6Y 3G73 3G8S 3G8T 3G96 3G9C 3G9Y 3GA1 3GA3 3GAU 3GAX 3GBJ 3GD8 3GDH 3GDX 3GF9 3GG6 3GGE 3GHG 3GHM 3GI0 3GJ0 3GJW 3GKJ 3GL6 3GLK 3GM3 3GOV 3GQC 3GQQ 3GR4 3GRO 3GV3 3GV5 3GV7 3GV8 3GYL 3H0H 3H1D 3H40 3H4B 3H4D 3H63 3H6N 3H7H 3H8K 3H8O 3H8Q 3H8R 3H8V 3H8X 3H8Z 3H91 3H95 3H9E 3H9Y 3HAJ 3HAK 3HBW 3HCS 3HD6 3HEQ 3HF1 3HFE 3HFH 3HFW 3HG3 3HHC 3HHD 3HHM 3HHN 3HI7 3HI9 3HIL 3HK0 3HKV 3HL2 3HLK 3HLT 3HM6 3HME 3HMI 3HMS 3HN3 3HNA 3HNC 3HNY 3HQA 3HQC 3HQI 3HR0 3HRN 3HRO 3HSH 3HTM 3HTU 3HU3 3HUP 3HW8 3HWN 3HWT 3HX0 3HX3 3HXO 3HXQ 3HY3 3HYG 3HYM 3HZJ 3I00 3I08 3I28 3I2B 3I2N 3I2V 3I33 3I35 3I3C 3I4A 3I4U 3I4W 3I5R 3I6C 3I6U 3I6X 3I84 3IA8 3IAI 3IAR 3IBJ 3ICU 3IDV 3IEI 3IEZ 3IF8 3IFA 3IGK 3IGL 3IH7 3IHJ 3IHR 3IHX 3II0 3II7 3IIJ 3IIN 3IJJ 3IKK 3IKL 3ILZ 3IN5 3IO2 3IOH 3IOL 3IQ2 3IQU 3IR3 3IRQ 3IRR 3ISB 3ISC 3ISD 3ISQ 3ITU 3IU1 3IU5 3IU6 3IUF 3IUG 3IUY 3IV1 3IVV 3IWL 3IWN 3IWP 3IX0 3IXS 3J0A 3J3D 3J3F 3JPN 3JPO 3JPP 3JPQ 3JPR 3JPS 3JPT 3JQH 3JUD 3JUI 3JUY 3JXF 3JXH 3JZY 3K05 3K0J 3K0W 3K1R 3K1W 3K1Z 3K26 3K2A 3K2J 3K2O 3K35 3K6G 3K6S 3K7I 3KAN 3KAT 3KB5 3KCI 3KDF 3KFV 3KG5 3KGR 3KGV 3KH0 3KHF 3KJD 3KJO 3KJP 3KMD 3KN1 3KN6 3KNB 3KNV 3KOV 3KQ0 3KQG 3KQI 3KQS 3KRM 3KS3 3KS9 3KSY 3KT9 3KTM 3KTU 3KTV 3KUP 3KUQ 3KUS 3KUZ 3KVH 3KVO 3KVQ 3KVW 3KW6 3KY9 3KZ8 3KZD 3L00 3L11 3L15 3L1X 3L2C 3L2P 3L3C 3L42 3L43 3L46 3L4C 3L4G 3L4H 3L4Y 3L50 3L5H 3L5I 3L5K 3L6A 3L6B 3L6X 3L81 3L9Q 3LCY 3LD6 3LE4 3LF5 3LFV 3LGD 3LH5 3LHR 3LJB 3LJU 3LJW 3LK9 3LL6 3LLH 3LLK 3LLM 3LLP 3LLU 3LM5 3LMN 3LNY 3LOF 3LPW 3LQ9 3LQH 3LQM 3LQV 3LRA 3LRE 3LRI 3LRN 3LRQ 3LRR 3LRU 3LS8 3LUC 3LUI 3LVR 3LWE 3LWK 3LXX 3LY5 3LYR 3LZB 3M03 3M06 3M1D 3M5B 3M66 3M7P 3M9J 3MAO 3MAX 3MAZ 3MB3 3MB4 3MBY 3MCB 3MCE 3MCF 3MDA 3MDC 3MDF 3MDG 3MDI 3MDM 3MEW 3MFK 3MGH 3MGI 3MIJ 3MJG 3MJK 3MK1 3MK4 3MK6 3MMY 3MNG 3MOP 3MOS 3MPX 3MQ4 3MQ7 3MQI 3MQL 3MQM 3MQP 3MR2 3MR3 3MR5 3MR6 3MSH 3MT5 3MTC 3MTR 3MTS 3MTT 3MU6 3MUJ 3MUM 3MUP 3MUR 3MUT 3MUV 3MVA 3MVB 3MWD 3MX7 3MXH 3MXN 3MXO 3MYI 3N00 3N01 3N2Z 3N3F 3N50 3N5N 3N6S 3N7Q 3N7S 3N8E 3N8I 3N9Y 3NA3 3NAF 3NAR 3NAU 3NBI 3NBN 3NCE 3NCL 3NCU 3NDD 3NDQ 3NER 3NEY 3NF1 3NGD 3NGO 3NGQ 3NH6 3NHC 3NHD 3NHE 3NHN 3NKB 3NKS 3NMD 3NMR 3NMW 3NMZ 3NNA 3NNC 3NNH 3NO8 3NOI 3NQJ 3NR1 3NR5 3NRX 3NSL 3NSZ 3NV1 3NVF 3NW0 3NWH 3NWN 3NWV 3NXA 3NXB 3NXP 3NXU 3NY3 3NY5 3NZL 3O0Z 3O10 3O22 3O2G 3O2T 3O36 3O3I 3O46 3O47 3O4R 3O5Q 3O6E 3O70 3O7V 3OA6 3OAD 3OB9 3OBQ 3OCP 3OD5 3OD8 3ODA 3ODC 3ODE 3ODW 3ODX 3OES 3OG6 3OG7 3OG8 3OGU 3OHU 3OLC 3OLJ 3OLL 3OMZ 3OOI 3OP3 3OP5 3OP8 3OPE 3ORH 3OSE 3OSK 3OSN 3OTC 3OU5 3OUI 3OV1 3OV6 3OVP 3OW8 3OX6 3P0C 3P0F 3P0L 3P0U 3P1A 3P1F 3P1J 3P1X 3P23 3P2T 3P3Y 3P49 3P4L 3P57 3P6D 3P6Y 3P7G 3P8C 3P8D 3PA6 3PB6 3PBH 3PC7 3PCV 3PD7 3PDF 3PDY 3PE0 3PE6 3PFF 3PFN 3PFS 3PFY 3PG6 3PG7 3PGW 3PH9 3PKI 3PLZ 3PM0 3PMI 3PML 3PMN 3PNC 3POW 3POZ 3PP2 3PPD 3PQ1 3PRY 3PS5 3PT3 3PTA 3PUA 3PUC 3PUF 3PV7 3PYC 3PZ7 3PZP 3Q01 3Q05 3Q06 3Q0H 3Q0L 3Q0M 3Q0N 3Q0O 3Q0P 3Q0Q 3Q0R 3Q0S 3Q13 3Q18 3Q1D 3Q1I 3Q2C 3Q2E 3Q2T 3Q2U 3Q6L 3Q6M 3Q6O 3Q6S 3Q6Z 3Q71 3Q72 3Q8K 3Q8L 3Q8M 3Q91 3Q93 3QCR 3QE2 3QE9 3QEA 3QEB 3QF2 3QFT 3QH9 3QI3 3QI5 3QII 3QIJ 3QIK 3QIR 3QIS 3QJ4 3QK3 3QKG 3QL9 3QLP 3QMB 3QMC 3QMD 3QMG 3QMH 3QMI 3QNT 3QO4 3QOW 3QP3 3QQN 3QRF 3QTE 3QU6 3QVE 3QWE 3QWL 3QWM 3QWP 3QWQ 3QX1 3QX3 3QXL 3QXR 3QXY 3QYE 3QYM 3QYN 3R0N 3R1H 3R1L 3R27 3R2P 3R3I 3R62 3R6B 3R6N 3R7G 3R8J 3R8Q 3R90 3R9A 3R9M 3RAU 3RAY 3RBG 3RBN 3RBS 3RC3 3RC8 3RCO 3RCP 3RCQ 3RCW 3RD2 3RDV 3RFE 3RGH 3RGK 3RH4 3RH5 3RH6 3RI4 3RIP 3RIY 3RJD 3RJE 3RJF 3RJG 3RJH 3RJI 3RJJ 3RJK 3RJO 3RK6 3RKQ 3RLE 3RLO 3RMU 3RN2 3RN5 3RNJ 3RNU 3RPP 3RPX 3RQ4 3RRQ 3RRU 3RSN 3RW6 3RY4 3RZ3 3RZG 3RZH 3RZJ 3RZK 3RZL 3RZM 3RZN 3RZV 3S24 3S4Y 3S57 3S58 3S59 3S5A 3S5J 3S5O 3S6W 3S79 3S7R 3S84 3S8I 3S8S 3S8W 3S92 3S93 3S94 3S95 3S98 3S9D 3S9G 3SAF 3SAK 3SC0 3SD6 3SEI 3SEN 3SF4 3SFJ 3SGM 3SGN 3SGO 3SGP 3SGR 3SGS 3SH4 3SHU 3SHW 3SI8 3SIU 3SIV 3SJM 3SKP 3SL9 3SM9 3SMJ 3SMQ 3SMT 3SMZ 3SNH 3SNV 3SOA 3SOC 3SOE 3SOM 3SOO 3SOV 3SP7 3SP8 3SPA 3SQD 3SR4 3SSU 3SW0 3SWK 3SWR 3SWY 3SWZ 3SYX 3SZA 3SZR 3T0H 3T0O 3T1I 3T1W 3T30 3T3L 3T5O 3T5X 3T6A 3T6P 3T7A 3T7L 3T92 3TBD 3TBG 3TC5 3TDC 3TDU 3TE3 3TEG 3TEQ 3TFR 3TFS 3TG4 3TGX 3THC 3THT 3THW 3THX 3THY 3THZ 3TIW 3TJM 3TJO 3TJQ 3TKU 3TKZ 3TLP 3TMI 3TMM 3TN2 3TNU 3TO8 3TOJ 3TOP 3TOW 3TQ1 3TQ6 3TRT 3TS8 3TSV 3TSZ 3TT0 3TT9 3TTJ 3TUO 3TV0 3TWR 3TYY 3TZD 3TZM 3U0R 3U0V 3U10 3U12 3U1K 3U1N 3U1U 3U21 3U23 3U2P 3U2U 3U3P 3U3Z 3U5L 3U5S 3U83 3U8I 3U9H 3U9J 3U9Q 3U9W 3UB2 3UBY 3UCG 3UCU 3UCW 3UCZ 3UD1 3UD3 3UD4 3UE2 3UEM 3UF1 3UFJ 3UFN 3UGC 3UI4 3UK3 3UKM 3ULH 3ULL 3UMH 3UN9 3UNN 3UO7 3UO9 3UOA 3UOB 3UOM 3UP1 3UPQ 3UQ0 3UQ2 3URF 3URO 3US0 3US1 3US2 3UUN 3UV2 3UV4 3UV5 3UVT 3UW5 3UWT 3UX2 3V2A 3V2B 3V30 3V33 3V34 3V3E 3V3L 3V42 3V43 3V4K 3V4O 3V4Q 3V53 3V56 3V70 3V79 3V8D 3V8S 3V98 3V9H 3VAF 3VAG 3VAH 3VAI 3VAJ 3VAL 3VAM 3VBB 3VD0 3VD1 3VD2 3VF3 3VFD 3VG7 3VHE 3VHS 3VHV 3VI6 3VJ9 3VKE 3VN9 3VNN 3VO3 3VOQ 3VOW 3VOY 3VPP 3VTU 3VTV 3VTW 3VVV 3VW9 3VYX 3VYY 3VZB 3W1B 3W3J 3W9Y 3ZCW 3ZD2 3ZDK 3ZI1 3ZIM 3ZJC 3ZJE 3ZN0 3ZNF 3ZNN 3ZNV 3ZON 3ZQK 3ZQS 3ZR0 3ZRH 3ZRT 3ZSJ 3ZTG 3ZVZ 3ZW5 3ZWF 3ZWT 3ZXF 3ZY0 3ZYQ 3ZYW 4A04 4A0D 4A0P 4A14 4A1G 4A1N 4A24 4A27 4A35 4A3N 4A3P 4A4F 4A4I 4A5S 4A5X 4A5Z 4A64 4A6D 4A7U 4A82 4A9C 4A9Z 4AA6 4AAA 4ABL 4ABM 4ACQ 4ACR 4AD9 4AE2 4AE7 4AE8 4AFL 4AGU 4AH6 4AIF 4AIW 4AJ5 4AJY 4AK8 4AKM 4AKV 4AL0 4ALG 4AMT 4ANK 4AOH 4AOW 4AP5 4AP8 4APO 4AQB 4AQL 4AS4 4ASC 4ASZ 4ATM 4AUV 4AVP 4AVS 4AW0 4AW6 4AWL 4AWN 4AY2 4AYA 4AYT 4AZ3 4AZ9 4B0F 4B2R 4B2S 4B3F 4B3G 4B4C 4B4O 4B53 4B5O 4B6D 4B6H 4B7L 4B7Y 4B87 4B91 4B94 4B9D 4BB9 4BBQ 4BC3 4BD2 4BDV 4BDX 4BEJ 4BGJ 4BGQ 4BHX 4BK0 4BKJ 4BKW 4BL1 4BN4 4BPB 4BQA 4BQY 4BSP 4D86 4D8K 4D8O 4D90 4DA1 4DA5 4DB1 4DBG 4DD8 4DDJ 4DDP 4DEQ 4DGJ 4DHX 4DIP 4DJC 4DK9 4DKC 4DKK 4DKX 4DL2 4DL3 4DL4 4DL5 4DL6 4DL7 4DLO 4DND 4DNL 4DO4 4DO9 4DOA 4DOB 4DOC 4DOH 4DON 4DOU 4DPZ 4DQY 4DRI 4DUR 4DVQ 4DWF 4DXT 4DY0 4DYL 4DYO 4DZO 4E1H 4E1I 4E1O 4E34 4E45 4E4H 4E54 4E5Y 4E5Z 4E6R 4E74 4E82 4E9E 4E9F 4E9G 4E9M 4EA4 4EA5 4EAR 4EBB 4EBC 4EBD 4EBE 4ECQ 4ECR 4ECS 4ECT 4ECU 4ECV 4ECW 4ECX 4ECY 4ECZ 4ED0 4ED1 4ED2 4ED3 4ED5 4ED6 4ED7 4ED8 4EEW 4EEY 4EF0 4EFO 4EGL 4EGX 4EHD 4EI1 4EI3 4EIH 4EJN 4EJQ 4EKU 4EKZ 4ELJ 4ELL 4EMO 4EMT 4ENZ 4EO7 4EOT 4EOZ 4EPU 4ERC 4ERN 4ERV 4ERY 4ES7 4ESR 4EUT 4EUU 4EUW 4EWE 4EWI 4EYH 4EYI 4EZF 4F02 4F0D 4F11 4F14 4F25 4F2J 4F3J 4F3T 4F6M 4F6N 4F6U 4F7H 4F7O 4F80 4F92 4F9C 4F9K 4F9Z 4FBN 4FC7 4FCJ 4FDI 4FGL 4FH0 4FHQ 4FIE 4FKA 4FKL 4FL3 4FLA 4FLB 4FMU 4FMW 4FNC 4FO0 4FO6 4FO9 4FOM 4FQG 4FQN 4FQP 4FR4 4FRW 4FTG 4FU3 4FU6 4FVQ 4FWW 4FXM 4FXV 4FXW 4FYO 4FYT 4FZV 4G0F 4G1M 4G1T 4G31 4G3O 4G82 4G83 4G84 4G85 4G8K 4G9A 4GA0 4GA7 4GBA 4GDK 4GDV 4GE6 4GEH 4GEI 4GGA 4GGC 4GGF 4GIF 4GIW 4GJZ 4GL2 4GLM 4GLP 4GMJ 4GMV 4GNE 4GO6 4GOF 4GOS 4GQ4 4GQB 4GQR 4GRZ 4GS4 4GT4 4GUT 4GV1 4GV2 4GWG 4GWM 4GXL 4GYW 4GYX 4H10 4H22 4H27 4H2D 4H2G 4H6Y 4H75 4H7W 4H7Y 4H87 4H9N 4HAE 4HAN 4HAS 4HBD 4HBQ 4HC4 4HC7 4HC9 4HCA 4HCK 4HCU 4HCZ 4HFX 4HL4 4HLH 4HOQ 4HOR 4HOS 4HOT 4HOU 4HPF 4HPM 4HQA 4HQU 4HQX 4HR9 4HRG 4HT2 4HTJ 4HTM 4HTP 4HVC 4HW4 4HWK 4HWN 4HXH 4HY4 4HZH 4HZR 4HZS 4I1F 4I4E 4I5I 4I5J 4I6O 4I6X 4I79 4IAX 4IC3 4IC7 4IDO 4IDT 4IE5 4IEJ 4IF8 4IG8 4IGD 4IGG 4IGZ 4II1 4IIM 4IJD 4IJX 4IKD 4IKP 4IM0 4IN0 4INC 4IQR 4IQY 4IR5 4IS1 4ITJ 4IU6 4IUL 4IVE 4IYP 4J0W 4J15 4J19 4J1Y 4J37 4J3M 4J5R 4J6G 4J8S 4J9K 4J9L 4J9M 4J9N 4J9O 4J9P 4J9Q 4J9R 4J9S 4JA8 4JGC 4JGT 4JHN 4JHS 4JIF 4JJ7 4JJH 4JK8 4JNC 4JNK 4JOI 4JOL 4JON 4JQF 4JSN 4JUY 4JV8 4JVH 4JWM 4JWN 4JXO 4K6J 4K92 4KA4 4KBL 4KFO 4KM5 4KNV 4KRF 4KSY 4KT1 4L0N 4L58 4L6E 4SKN 5ZNF 6PAX 6RLX 7ICE 7ICF 7ICG 7ICH 7ICI 7ICJ 7ICK 7ICL 7ICM 7ICN 7ICO 7ICP 7ICQ 7ICR 7ICS 7ICT 7ICU 7ICV 8ICA 8ICB 8ICC 8ICE 8ICF 8ICG 8ICH 8ICI 8ICJ 8ICK 8ICL 8ICM 8ICN 8ICO 8ICP 8ICQ 8ICR 8ICS 8ICT 8ICU 8ICV 8ICW 8ICX 8ICY 8ICZ 9ICA 9ICB 9ICC 9ICE 9ICF 9ICG 9ICH 9ICI 9ICJ 9ICK 9ICL 9ICM 9ICN 9ICO 9ICP 9ICQ 9ICR 9ICS 9ICT 9ICU 9ICV 9ICW 9ICX 9ICY

**Supplementary Table 2.** Residues of DPP4 closest to the bound ligand with possible hydrogen bonds. Interactions sorted based on the distance. N: Number of atoms in the ligand, R/A/LA/D: Residue number/Atom of the residue/Atom of ligand/distance between the interacting atoms (in Å). For example, ‘E205/OE1/N25/2.7’ means that the atom OE1 from Glu205 is at 2.7 Å from the N25 atom of W94 in PDBid:3VJLA. For uniformity, we choose the first four closest atoms. This might result in choosing some atoms which are unlikely to form a hydrogen bond (for example, in PDBid:4J3JA S209/OG is at 4.8 Å from NAQ).

PDB	HET	N	R/A/LA/D	R/A/LA/D	R/A/LA/D	R/A/LA/D
3VJLA	W94	33	E205/OE1/N25/2.7	E206/OE1/N25/2.8	N710/ND2/O33/2.9	Y662/OH/O33/3
2AJ8A	SC3	26	E205/OE2/N13/2.7	E206/OE1/N13/3	Y631/N/O23/3.1	Y547/OH/N7/3.4
2RGUA	356	35	E205/OE2/N27/3	Y662/OH/N27/3.1	Y631/N/O10/3.1	E206/OE2/N27/3.1
4A5SA	N7F	37	E205/OE2/N18/2.7	E206/OE2/N18/2.7	Y662/OH/N18/2.8	Y631/N/O26/3
2QTBA	474	32	E205/OE2/N6/2.7	N710/ND2/O7/2.8	E206/OE1/N6/2.8	R358/NE/N56/2.8
2OGZA	U1N	24	Y631/N/O25/3	E206/OE1/N12/3.1	R125/NH2/O15/3.1	E205/OE2/O15/3.3
2JIDA	GVB	24	E206/OE2/N20/2.8	E205/OE2/N20/2.9	Y662/OH/N20/3	R125/NH1/O25/3.8
2I78B	KIQ	31	Y662/OH/N/3	E205/O/O/4.1	E206/OE1/O/4.2	R669/NH2/O/4.4
3H0CA	PS4	32	E205/OE2/N21/2.7	Y662/OH/N21/2.9	E206/OE2/N21/2.9	Q553/N/O3/3
2AJLI	JNH	24	S630/OG/N3/2.3	E206/OE2/N2/2.5	Y547/OH/N3/2.6	E205/OE2/N2/2.7
2BUBA	FPB	28	E206/OE2/N18/2.5	E205/OE2/N18/2.9	Y662/OH/N18/3	Y547/OH/O16/4.3
4DSAA	D1C	29	E206/OE2/NAY/2.6	E205/O/OBC/3.1	Y662/OH/NAY/3.1	Y585/OH/NAI/3.9
2OPHA	277	23	E205/OE2/N33/2.7	N710/ND2/O32/2.8	Y662/OH/N33/3.1	E206/OE2/N33/3.1
1RWQA	5AP	27	Y662/OH/N21/2.5	E206/OE2/N21/2.8	E205/OE2/N23/2.9	R125/NH2/N1/3.4
2QJRA	PZF	29	E205/OE2/N20/2.6	R358/NE/O18/2.8	E206/OE2/N20/2.9	Y662/OH/N20/3
2FJPA	S14	31	E205/OE2/N30/2.7	N710/ND2/O32/2.8	E206/OE2/N30/2.8	Y547/OH/O33/2.8
2OAEA	AIL	21	E203/OE2/N2/2.8	E204/OE2/N2/2.8	N711/ND2/O8/3.1	Y663/OH/N9/3.1
3G0CA	RUF	27	E205/OE1/N9/3	E206/OE1/N9/3.2	Y631/N/O23/3.4	Y547/OH/N12/3.6
3C43A	315	31	E205/OE2/N6/2.8	Y662/OH/N6/3	N710/ND2/O5/3	E206/OE2/N6/3
3BJMA	BJM	23	S630/OG/N23/2.4	E205/OE2/N7/2.7	E206/OE2/N7/2.7	Y547/OH/O15/2.8
3O95A	01T	26	E206/OE2/N13/2.5	E205/OE1/N13/2.8	Y662/OH/N13/2.8	R125/NH1/O19/3
3G0GA	RUM	24	E205/OE1/N24/2.9	E206/OE1/N24/3.1	Y631/N/O8/3.2	R125/NH2/N17/3.3
2G5PA	ADF	29	S630/OG/N22/2.4	Y662/OH/N8/3.1	Y547/OH/N22/3.1	E206/OE2/N7/3.1
2BUCA	008	26	E206/OE2/N10/2.7	Y662/OH/N10/2.8	E205/OE2/N10/3	Y547/OH/O13/4.5
2QOEA	448	29	E206/OE2/N20/2.7	E205/OE2/N20/2.9	Y662/OH/N20/2.9	Y547/OH/O22/4.6
2OLEA	KR2	30	E206/OE2/NAM/2.7	Y662/OH/NAM/3.6	E205/OE2/NAM/4	Y547/OH/OAP/4.5
3KWFA	B1Q	27	E205/OE2/N21/2.7	N710/ND2/O19/2.7	Y662/OH/N21/3	R125/NH2/O19/3
3SX4A	KXA	58	Y662/OH/N25/2.7	E206/OE2/N25/2.7	E205/OE2/N25/2.8	R125/NH1/O26/3.1
2ONCA	SY1	27	E205/OE1/N1/2.6	Y631/N/O17/3.1	Y547/OH/N18/3.2	E206/OE1/N1/3.4
2I03B	AXD	29	S630/OG/N14/2.4	E206/OE1/N1/2.8	Y662/OH/O16/2.9	Y547/OH/N14/3
3KWJA	23Q	27	E205/OE2/N17/2.6	Y662/OH/N17/2.8	E206/OE2/N17/2.8	S209/OG/O19/3.3
3CCCA	7AC	21	E205/OE1/N20/2.5	Y662/OH/N20/2.7	E206/OE2/N20/3.2	Y631/N/N9/3.3
3SWWA	KXB	25	E205/OE2/N21/2.7	Y662/OH/N21/2.8	E206/OE2/N21/2.9	R125/NH2/N19/3.5
4G1FA	0WG	24	E206/OE2/N9/2.8	Y662/OH/N9/2.9	Y547/OH/N2/3.1	Y631/N/O20/3.1
3C45A	317	30	E205/OE2/N6/2.8	E206/OE2/N6/2.8	Y662/OH/N6/3	Y547/OH/N29/3.7
2G63B	AAF	29	S630/OG/N18/2.4	E205/OE2/N7/2.6	Y662/OH/N8/3.1	Y547/OH/N18/3.1
1X70A	715	28	E206/OE2/N20/2.7	E205/OE2/N20/2.8	Y662/OH/N20/2.8	S209/OG/N27/3.9
2GBIA	XIH	29	E204/OE2/N14/2.3	Y632/N/O/2.8	E203/OE2/N14/3	Y663/OH/N14/3.1
3G0BA	T22	25	E205/OE1/N13/2.5	R125/NH2/N24/3.1	Y631/N/O26/3.2	E206/OE1/N13/3.3
2IITA	872	28	E205/OE2/N20/2.7	Y662/OH/N20/2.8	E206/OE2/N20/2.9	N710/OD1/N20/4.5
4JH0A	1MD	27	Y662/OH/N16/2.7	E206/OE2/N16/2.7	Y547/OH/O1/2.8	E205/OE2/N16/2.9
4LKOA	1WH	25	Y662/OH/N/2.7	E206/OE2/N/2.8	E205/OE2/N/2.9	Y547/OH/O2/3
2RIPA	34Q	25	N710/ND2/O1/2.6	E205/OE2/N3/2.8	Y662/OH/N3/2.8	E206/OE1/N3/2.8
3QBJA	NXZ	25	Y662/OH/N18/2.7	E206/OE2/N18/2.7	N710/ND2/O25/2.8	E205/OE2/N18/2.9
3HACA	361	23	Y662/OH/N23/2.7	E205/OE2/N23/2.8	E206/OE1/N12/4.2	N710/OD1/N23/4.3
3VJMA	W61	32	E205/OE1/N28/2.7	E206/OE1/N28/2.7	Y662/OH/O57/2.9	N710/ND2/O57/2.9
3O9VA	10T	23	Y547/OH/O15/2.5	E206/OE2/N19/2.6	Y662/OH/N19/2.7	E205/OE1/N19/2.8
4DSZA	DC3	26	E206/OE2/NAM/2.8	E205/OE2/NAM/3.1	Y662/OH/NAM/3.1	S209/OG/NAR/4.7
4J3JA	D3C	30	E206/OE2/NAM/2.8	E205/OE2/NAM/3.1	Y662/OH/NAM/3.3	S209/OG/NAQ/4.8
4DTCA	D5C	33	E206/OE2/NAM/2.7	E205/OE2/NAM/3.1	Y662/OH/NAM/3.3	R669/NH2/OAQ/4.2
3OPMA	LUI	28	E205/OE1/N18/2.7	Y662/OH/N18/2.8	E206/OE2/N18/2.9	W629/O/N27/3
2OAGB	DLI	31	E205/OE2/N22/2.5	Y662/OH/N22/2.6	E206/OE2/N22/2.9	R358/NE/O1/3.2
2GBGA	1AD	19	S631/OG/N12/2.4	E203/OE2/N14/2.7	Y548/OH/N12/3	E204/OE2/N14/3.1
2HHAA	3TP	26	E205/OE2/N6/2.7	E206/OE2/N6/2.7	Y662/OH/N6/2.9	N710/ND2/O5/2.9
2QT9A	524	31	E205/OE2/N19/2.6	E206/OE2/N19/2.8	Y662/OH/N19/2.9	N710/ND2/O20/2.9
2OQVA	MA9	32	E206/OE2/N27/2.7	Y662/OH/N27/3	R358/NE/O4/3.1	E205/OE2/N27/3.3
3F8SA	PF2	26	E205/OE2/N3/2.5	Y662/OH/O7/2.8	N710/OD1/O7/3	E206/OE2/N3/3.2
2AJBA	0QG	24	S630/OG/N2/2.4	E205/OE2/N/2.7	HIS740/NE2/O2/2.9	Y662/OH/O/3
2G5TA	ACF	26	S630/OG/N22/2.4	E205/OE2/N7/2.6	Y662/OH/O3/3	N710/ND2/O3/3
3NOXA	6A5	28	E205/OE2/N16/2.4	E206/OE2/N16/2.7	Y662/OH/N16/3	R125/NH2/N4/3.7
3W2TA	LF7	22	S630/OG/N2/2.4	E205/OE1/N12/2.8	Y662/OH/O20/3	E206/OE2/N12/3
3D4LA	605	26	E205/OE2/N15/2.6	R358/NE/O42/2.8	Y662/OH/N15/2.9	V207/O/N41/2.9
2QKYA	13Z	26	S630/OG/O2/2.1	E205/O/O4/2.5	Y547/OH/O2/2.6	E206/OE1/O4/2.8
3Q8WA	AZV	38	R125/NH1/O/2.5	E206/OE2/NAG/2.6	Y662/OH/NAG/2.8	E205/OE2/NAG/2.9
3EIOA	AJH	33	Y585/OH/OBD/2.6	E205/OE2/NBG/2.7	Y662/OH/NBG/2.9	E206/OE2/NBG/2.9
3Q0TA	LGE	26	Y662/OH/N21/2.6	E205/OE2/N21/2.7	E206/OE2/N21/2.9	R125/NH1/O22/3.4
2P8SA	417	58	E205/OE2/N38/2.8	E206/OE2/N38/2.9	Y662/OH/N38/3.2	S209/OG/N34/3.4
2OQIB	GGO	28	E205/OE2/N/2.4	Y662/OH/N/2.7	E206/OE2/N/2.9	R358/NE/O/3.2
4PNZA	2VH	28	E205/OE2/N/2.7	E206/OE2/N/2.8	Y662/OH/N/2.9	Y547/OH/O/3.2
3VJKA	M51	30	E205/OE1/N21/2.9	Y662/OH/O30/2.9	E206/OE1/N21/2.9	N710/ND2/O30/3
3OC0A	B2Q	23	E205/OE2/NS/2.8	S209/OG/OB/2.9	Y662/OH/NS/3.4	E206/OE2/NS/3.5
4N8DA	2KS	24	E206/OE2/N10/2.7	E205/OE2/N10/2.8	Y662/OH/N10/2.8	N710/OD1/N10/4.5
4N8EA	2KV	22	E205/OE2/N15/2.7	E206/OE2/N15/2.7	Y662/OH/N15/2.8	N710/OD1/N15/4.3
2IIVA	565	24	E205/OE2/N20/2.7	Y662/OH/N20/2.8	E206/OE2/N20/2.9	N710/ND2/N20/4.4
3HABA	677	27	E205/OE2/N23/2.7	Y662/OH/N23/2.7	E206/OE1/N12/4.3	N710/OD1/N23/4.4
2I3ZA	LIR	27	E203/OE2/N18/2.5	Y632/N/O9/2.7	Y548/OH/N6/3.4	E204/OE2/N18/3.4

**Supplementary Table 3.** Library of non-redundant motifs. This library of motifs can be used to query any protein using CLASP to determine the possibility that DPP4 inhibitors might bind to it.

PDB	Motif Name	Motif
3VJLA	2OQVA1	GLU205/OE1 GLU206/OE1 TYR662/OH ASN710/ND2
2AJ8A	2OQVA2	GLU205/OE2 GLU206/OE1 TYR547/OH TYR631/N
2RGUA	2OQVA3	GLU205/OE2 GLU206/OE2 TYR631/N TYR662/OH
2QTBA	2OQVA4	GLU205/OE2 GLU206/OE1 ARG358/NE ASN710/ND2
2OGZA	2OQVA5	ARG125/NH2 GLU205/OE2 GLU206/OE1 TYR631/N
2JIDA	2OQVA6	ARG125/NH1 GLU205/OE2 GLU206/OE2 TYR662/OH
2I78B	2OQVA7	GLU205/O GLU206/OE1 TYR662/OH ARG669/NH2
3H0CA	2OQVA8	GLU205/OE2 GLU206/OE2 GLN553/N TYR662/OH
2AJLI	2OQVA9	GLU205/OE2 GLU206/OE2 TYR547/OH SER630/OG
2BUBA	2OQVA10	GLU205/OE2 GLU206/OE2 TYR547/OH TYR662/OH
4DSAA	2OQVA11	GLU205/O GLU206/OE2 TYR585/OH TYR662/OH
2OPHA	2OQVA12	GLU205/OE2 GLU206/OE2 TYR662/OH ASN710/ND2
1RWQA	2OQVA13	ARG125/NH2 GLU205/OE2 GLU206/OE2 TYR662/OH
2QJRA	2OQVA14	GLU205/OE2 GLU206/OE2 ARG358/NE TYR662/OH
2FJPA	2OQVA15	GLU205/OE2 GLU206/OE2 TYR547/OH ASN710/ND2
2OAEA	2OQVA16	GLU205/OE2 GLU206/OE2 TYR662/OH ASN711/ND2
3G0CA	2OQVA17	GLU205/OE1 GLU206/OE1 TYR547/OH TYR631/N
3O95A	2OQVA18	ARG125/NH1 GLU205/OE1 GLU206/OE2 TYR662/OH
3G0GA	2OQVA19	ARG125/NH2 GLU205/OE1 GLU206/OE1 TYR631/N
2G5PA	2OQVA20	GLU206/OE2 TYR547/OH SER630/OG TYR662/OH
3KWFA	2OQVA21	ARG125/NH2 GLU205/OE2 TYR662/OH ASN710/ND2
2I03B	2OQVA22	GLU206/OE1 TYR547/OH SER630/OG TYR662/OH
3KWJA	2OQVA23	GLU205/OE2 GLU206/OE2 SER209/OG TYR662/OH
3CCCA	2OQVA24	GLU205/OE1 GLU206/OE2 TYR631/N TYR662/OH
4G1FA	2OQVA25	GLU206/OE2 TYR547/OH TYR631/N TYR662/OH
2G63B	2OQVA26	GLU205/OE2 TYR547/OH SER630/OG TYR662/OH
2IITA	2OQVA27	GLU205/OE2 GLU206/OE2 TYR662/OH ASN710/OD1
2RIPA	2OQVA28	GLU205/OE2 GLU206/OE1 TYR662/OH ASN710/ND2
3HACA	2OQVA29	GLU205/OE2 GLU206/OE1 TYR662/OH ASN710/OD1
3O9VA	2OQVA30	GLU205/OE1 GLU206/OE2 TYR547/OH TYR662/OH
4DTCA	2OQVA31	GLU205/OE2 GLU206/OE2 TYR662/OH ARG669/NH2
3OPMA	2OQVA32	GLU205/OE1 GLU206/OE2 TRP629/O TYR662/OH
2AJBA	2OQVA33	GLU205/OE2 SER630/OG TYR662/OH HIS740/NE2
2G5TA	2OQVA34	GLU205/OE2 SER630/OG TYR662/OH ASN710/ND2
3W2TA	2OQVA35	GLU205/OE1 GLU206/OE2 SER630/OG TYR662/OH
3D4LA	2OQVA36	GLU205/OE2 VAL207/O ARG358/NE TYR662/OH
2QKYA	2OQVA37	GLU205/O GLU206/OE1 TYR547/OH SER630/OG
3EIOA	2OQVA38	GLU205/OE2 GLU206/OE2 TYR585/OH TYR662/OH
2I3ZA	2OQVA39	GLU205/OE2 GLU206/OE2 TYR547/OH TYR631/N

## Supplementary information

Supplementary Pymol scripts. Click here to access the files. http://dx.doi.org/10.5256/f1000research.3002.s40929
