# Supplementary material for: The dipeptidyl peptidase IV inhibitors vildagliptin and K-579 inhibit a phospholipase C: a case of promiscuous scaffolds in proteins
Source: F1000Res. 2015 Jan 29;2:286. Originally published 2013 Dec 27. [Version 3] doi: 10.12688/f1000research.2-286.v3 (PMC4309170; doi:10.12688/f1000research.2-286.v3)
Supplement: Peer review history

# Peer review: Referee response for version 2

**DOI:** 10.5256/f1000research.6371.r7383

**Published:** 2015-01-20

**Authors:** Mark D Gorrell

**Affiliations:** 1 Molecular Hepatology, Centenary Institute, Newtown, NSW, Australia

Thank you to the authors for developing this paper.

I have some further comments.

 

1. The primary issue now is the speculation in the title.
  The title seeks to extrapolate the obtained data on two compounds to suggest that it is applicable to all DPP-IV inhibitors.
  That is, the speculation of this paper is that the presented data is relevant to an entire drug class. The comments and the title should be restricted to one or two of the compounds that were studied in this paper. Moreover, K-579 is not a diabetes drug. In this context, the title needs changing to avoid ambiguity.
  I suggest:
  “The dipeptidyl peptidase IV inhibitor vildagliptin used in type 2 diabetes inhibits a phospholipase C: a case of promiscuous scaffolds in proteins.”
  or
  “The dipeptidyl peptidase IV inhibitors vildagliptin and K-579 inhibit a phospholipase C: a case of promiscuous scaffolds in proteins.”
2. This study complements the much broader work using focused, direct technology for measuring and detecting off-target inhibition. That paper is published in Nature Chemical Biology in 2014 ( Bachovchin *et al.* 2014). That study similarly showed that vildagliptin inhibits enzymes other than DPP-IV. That study showed that DPP4 inhibitors differ, such that sitagliptin does not inhibit other enzymes.
  The authors need to comment and restrict their conclusions to the compounds that they studied rather than imply that DPP-IV inhibiting compounds that they did not study, such as sitagliptin, have similar characteristics to the compounds that they did study.
3. The data of this study is biochemical yet 16 of the cited references concern the safety of DPP-IV inhibition. The manuscript now carefully does not draw a link to drug safety; the title needs to do the same.
4. As the paper is focused upon DPP-IV structure and function, more papers on this topic could be cited and linked with the data. For example, the author's amendment mentions contacts in DPP-IV at Glu205, Glu206 and Tyr662. The authors could state that Glu205 and Glu206 have been shown to be essential for catalysis by DPP-IV and cite the paper Abbott *et al.* (1999).

I have read this submission. I believe that I have an appropriate level of expertise to confirm that it is of an acceptable scientific standard.

# Response

**Published:** 2015-01-28

**Authors:** Sandeep Chakraborty

**Affiliations:** Tata Institute of Fundamental Research, India

We would like to thank you for your positive comments, and your informative suggestions.

We agree with your suggested change in the title. In the latest version, we have also cited the research you have brought to our attention.

# Peer review: Referee response for version 1

**DOI:** 10.5256/f1000research.3236.r4249

**Published:** 2014-03-26

**Authors:** Mark D Gorrell

**Affiliations:** 1 Molecular Hepatology, Centenary Institute, Newtown, NSW, Australia

The successful targeting of DPP4 using small molecule compounds to treat type 2 diabetes has attracted a great deal of attention towards the study of this protease.

The authors applied sophisticated techniques that they have developed in order to discover that two DPP4 inhibitors, including one that is in limited clinical use, can to some extent inhibit the activity of a bacterial lipase (PI-PLC). Many lipases and esterases and hydrolases including DPP4 and related enzymes use the *alpha/beta* hydrolase fold and the authors show how this related protein topology can place the residues in positions that are sufficiently similar to interact with an inhibitor. 

The major difficulty with this paper is that it attempts to connect these data with possible clinical outcomes. No evidence for such a link is presented. Therefore, the title and much of the conclusions need to be modified so that they reflect the data without speculation.

Two inhibitors of DPP4, LAF237 and K-579, were studied. K-579 is not in clinical use. LAF237 is licensed in Europe and is known to exhibit some inhibition of the DPP4-related proteases DPP8 and DPP9. The extent of inhibition of DPP8 and DPP9 by LAF237 is believed not to have physiological effects in humans. The IC50 of LAF237 on DPP9 is less than 0.01 mM. The IC50 of LAF237 on bacterial PI-PLC is 0.1mM, which is close to the lower limit of detection of inhibition of an enzyme. No mammalian homolog of PI-PLC was examined.

The literature that the authors cite to suggest that DPP4 inhibition might be detrimental for human health, particularly the pancreas, is data on sitagliptin or exenatide. Exenatide is not a DPP4 inhibitor and sitagliptin is quite different to LAF237, both in protease specificity and in chemical structure. The contact points of LAF237 and sitagliptin in the catalytic site of DPP4 differ considerably. The authors present no data on sitagliptin or any other DPP4 inhibitor (other than LAF237) that in is the clinic.

The images of overlaid catalytic triads of various enzymes presented in Fig 1 and Fig 3 need to be depicted in 3D in order to evaluate how close they are in 3D. Intermolecular distances should be shown on thee figures. To convince the reader that LAF237 sits into and makes contacts with enzymes other than DPP4, we need to see the compound docked into the structure of each enzyme of interest.

I have read this submission. I believe that I have an appropriate level of expertise to confirm that it is of an acceptable scientific standard, however I have significant reservations, as outlined above.

# Response

**Published:** 2014-12-10

**Authors:** Sandeep Chakraborty

**Affiliations:** Tata Institute of Fundamental Research, India

We would like to thank you for taking the time to review this paper, and also for your insightful comments. We also apologize for the inordinate time taken to respond to your comments. A lot of this time was spent in understanding docking methods, instead of blindly applying this to the problem at hand. A by-product of this learning process was the implementation of a new method (DOCLASP) for docking molecules to proteins ^1^. We have docked vildagliptin to the PI-PLC structure complexed with myo-inositol using DOCLASP. Based on your suggestion, we have also

done a comprehensive analysis of all 76 known DPP4 structures liganded to inhibitors till date.

Please find out detailed responses to your comments below.

- *The successful targeting of DPP4 using small molecule compounds to treat type 2 diabetes has attracted a great deal of attention towards the study of this protease. The authors applied sophisticated techniques that they have developed in order to discover that two DPP4 inhibitors, including one that is in limited clinical use, can to some extent inhibit the activity of a bacterial lipase (PI-PLC). Many lipases and esterases and hydrolases including DPP4 and related enzymes use the alpha/beta hydrolase fold and the authors show how this related protein topology can place the residues in positions that are sufficiently similar to interact with an inhibitor. The major difficulty with this paper is that it attempts to connect these data with possible clinical outcomes. No evidence for such a link is presented. Therefore, the title and much of the conclusions need to be modified so that they reflect the data without speculation.*
  We have tried to keep away from taking sides on the clinical outcomes, since that is not our forte. Also, we believe our title is innocuous in that context - it just speaks of promiscuous scaffolds. We only highlight that if (and only if) our data of PIPLC inhibition holds true for human lipases, then it might provide some arguing points for those worried about the side effects of these drugs.
  For example, we say ‘The reported elevated levels of serum lipase, although contested, could be rationalized by inhibition of lipases reported here’. If you could kindly point out specifically any speculations that is unwarranted, we will modify those.
- *Two inhibitors of DPP4, LAF237 and K-579, were studied. K-579 is not in clinical use. LAF237 is licensed in Europe and is known to exhibit some inhibition of the DPP4-related proteases DPP8 and DPP9. The extent of inhibition of DPP8 and DPP9 by LAF237 is believed not to have physiological effects in humans.*
  Since this study does not emphasize on the clinical relevance of the inhibitions (but on the methodology of finding such interactions), and we are not a group specializing in diabetes, we believe the choice of the inhibitors would not alter our reasoning our conclusions.
- *The IC50 of LAF237 on DPP9 is less than 0.01 mM. The IC50 of LAF237 on bacterial PI-PLC is 0.1mM, which is close to the lower limit of detection of inhibition of an enzyme.*
  We agree to this point. However, K-579 was inhibiting even at nanomolar concentrations.
- *No mammalian homolog of PI-PLC was examined.*
  We are currently evaluating that possibility.
- *The literature that the authors cite to suggest that DPP4 inhibition might be detrimental for human health, particularly the pancreas, is data on sitagliptin or exenatide. Exenatide is not a DPP4 inhibitor and sitagliptin is quite different to LAF237, both in protease specificity and in chemical structure.*
  We were referring to the inhibitor part of the data, but that point needs to be made explicit as you have correctly pointed out. Also, we agree that the possible difference of sitagliptin with LAF237 needs to be stated. We have modified the text to include these criticisms. Once again, we reiterate we intend not to comment on clinical outcomes or debates, but to suggest a rational methodology to act as a guide for tests that look for possible interactions.
- *The contact points of LAF237 and sitagliptin in the catalytic site of DPP4 differ considerably. The authors present no data on sitagliptin or any other DPP4 inhibitor (other than LAF237) that in is the clinic.*
  We have included a comprehensive study on the contact points of various inhibitors. Once again, this does not negate any of our conclusions.
- *The images of overlaid catalytic triads of various enzymes presented in Fig 1 and Fig 3 need to be depicted in 3D in order to evaluate how close they are in 3D.*
  The 3D images of the superimposition of these enzymes are not pleasing to the eye, since they lack structural homology. However, we have added a PyMol script in case someone wishes to do that (Superimposeproteins.p1m). The script specifies the color coding of the residues.
- *Intermolecular distances should be shown on thee figures.*
  Once again, we think that the intermolecular distances clutter the figure. The superimposition gives an approximate idea of the congruence. The exact values are specified in Table 1. We have modified the legend of Fig.3 to specify that.
- *To convince the reader that LAF237 sits into and makes contacts with enzymes other than DPP4, we need to see the compound docked into the structure of each enzyme of interest.*
  As mentioned previously, we have docked sitagliptin to PI-PLC using DOCLASP ^1^. We have provided the Pymol script as supplementary data to help visualize the docking. There is no solved structure where LAF237 inhibits DPP4.

Once again, we are thankful for the comments. We hope that we have addressed your concerns by the changes that we have made, and that the manuscript will be found suitable in the modified form.

**References**

1. Chakraborty S. *DOCLASP* - Docking ligands to target proteins using spatial and electrostatic congruence extracted from a known holoenzyme and applying simple geometrical transformations [v2; ref status: awaiting peer review, http://f1000r.es/4pb] *F1000Research* 2014, **3**:262 (doi: 10.12688/f1000research.5145.2)

# Peer review: Referee response for version 1

**DOI:** 10.5256/f1000research.3236.r3818

**Published:** 2014-03-06

**Authors:** Rodney Rouse

**Affiliations:** 1 Division of Applied Regulatory Science, Center for Drug Evaluation and Research, U.S. Food and Drug Administration, Silver Spring, MD, USA

**Disclaimer: I lack the protein chemistry expertise to comment on the assumptions and protein chemistry used in the computational method described in this article.**

The title and abstract are appropriate.The overall experimental design is simple but strong and well suited for this project. The methods were generally well described. The conclusions are not overstated and any implications are justified based on the presented data. The article is very well written.   

This is a very interesting study that uses a previously defined computational method, CataLytic Active Site Prediction (CLASP), that compares structural and charge similarities of catalytic sites to identify functionally similar proteins. This methodology was used to assess the potential for adverse events based on off target effects of the inhibitors of DPP-IV. Using CLASP, the authors had previously indentified a Bacillus cerus phosphoinositide specific phospholipase-C (PI-PLC) as similar in active catalytic site to the enzyme, DPP-IV. They used laboratory techniques to verify this finding. 

In the present study, the authors demonstrated the ability of two separate DPP-IV inhibitors to significantly reduce the activity of this PI-PLC in the lab. Subsequent to this experimentation, the authors returned CLASP to identify catalytic sites in other proteins that might also be inhibited by DPP-IV inhibitors thereby yielding unforeseen inhibition and biological effects. As applied to the case of DPP-IV inhibitors, which are not extremely specific, the authors identify a number of other proteins that could be promiscuously impacted by DPP-IV inhibitors thereby providing mechanisms for unexpected adverse events. Although the significance of DPP-IV inhibitor related adverse events has yet to be determined, the fact that changes have been reported non-clinically and clinically are undeniable. Eventually, the benefit of these molecules may far outweigh their associated risks, but the authors provide a potential path forward for investigation of unexpected events with this class of drug. If contradictory reports persist, this path may require further illumination. 

The approach is theoretically similar to using structural similarities to identify off target receptor binding and consequent biological effects, an expanding approach in safety assessment and in identification of mechanisms for adverse events in the pharmaceutical lifecycle. Similarly, this method could be predictive for off target effects and suggest what those effects might be. However, whether this is a method that can be generally applicable to other molecules is beyond my ability to comment and the scope of this work.

 Comments/Suggestions:

1) Were the inhibition experiments done in duplicate, triplicate, etc?  Some slight expansion of the protocols would help with attempts to replicate.

I have read this submission. I believe that I have an appropriate level of expertise to confirm that it is of an acceptable scientific standard.

# Response

**Published:** 2014-03-10

**Authors:** Adela Rendón-Ramirez

**Affiliations:** Unidad de Biofisica CSIC UPV, Spain

Dear Dr Rouse,

We would like to thank you for taking the time and reviewing our paper. Your positive comments encourage us to further our research in this area.

We concur with your statement - *"Eventually, the benefit of these molecules may far outweigh their associated risks"*. And it is our endeavor to improve the accuracy and generality of our method through different compounds. We would specifically like to highlight another case of antagonist binding identified through CLASP, although in this case most alkaline phosphatases were not affected - Chakraborty *et al.*(2012)

The data for PI-PLC inhibition using DPP4 inhibitors, as shown in Figure 2, are average values of two closely similar experiments. We will revise the manuscript to include this point when we hear from another referee.

Best regards,

Sandeep Chakraborty and Adela Rendón-Ramirez
